# Temporal characterisation and electrophysiological implications of TBI-induced serine/threonine kinase activity in mouse cortex

**DOI:** 10.1007/s00018-025-05638-4

**Published:** 2025-03-05

**Authors:** Celine Gallagher, Thomas Mittmann

**Affiliations:** Institute of Physiology, University Medical Centre of the Johannes Gutenberg University Mainz, Mainz, Germany

**Keywords:** Kinase, Kinome, Traumatic brain injury, Hyperexcitability, Phosphorylation, Cortical networks

## Abstract

Traumatic brain injury (TBI) remains the leading cause of death and disability worldwide with no existing effective treatment. The early phase after TBI induction triggers numerous molecular cascades to regulate adaptive processes and cortical network activity. Kinases play a particularly prominent role in modifying peptide substrates, which include ion channels, receptors, transcription factors and inflammatory mediators. This study aimed to better understand the post-injury serine/threonine kinome; (1) Which kinases conduct phosphorylation-induced alterations of target peptides following unilateral TBI in mouse cortex? (2) How do these kinases effectuate pathological network hyperexcitability, which has detrimental long-term outcomes? We used a serine/threonine kinase assay at 4 h, 24 h and 72 h post-TBI to identify hyper-/hypo-active/phosphorylated kinases and peptides in the ipsilateral and contralateral cortical hemispheres relative to sham-operated controls. We pharmacologically mimicked the changes seen in ERK1/2 and PKC kinase activity, and using microelectrode array recordings we explored their significant electrophysiological implications on spontaneous and evoked cortical activity. We then used these findings to manipulate key kinase activity changes at 24 h post-TBI to rescue the hyperexcitability that is seen in the contralateral cortical network at this timepoint back to sham level. The contribution of specific downstream peptide target channel/receptor subunits was also shown. We conclude that volatile kinase activity has potent implications on cortical network activity after the injury and that these kinases and/or their peptide substrates should be more seriously considered as therapeutic targets for the clinical treatment of TBI.

**Supplementary Information:**

The online version contains supplementary material available at 10.1007/s00018-025-05638-4.

## Introduction

Traumatic brain injury (TBI) remains a debilitating and prevalent neurological disorder that still affects tens of millions of people annually, despite decades of in-depth research [1–4]. Effective therapeutic treatment for TBI is still lacking, and the currently employed treatment strategies unfortunately leave much room for advancement [5, 6]. To treat TBI adequately is a challenge for a plethora of reasons, but perhaps the most pronounced of these is that TBI presents a rich interpatient pathoheterogeneity [7, 8]. These problems can compound as time transpires; the diverse chronic effects of TBI can be very impactful on the patient, especially when considering quality of life and general comfort and wellbeing [9–11].

TBI can be broken down into the primary injury and the secondary injuries [12]. The primary injury consists of the direct and instantaneous effects of the brain trauma; the harm the impact immediately causes, e.g., necrosis of the neuronal cells in the lesion centre, disruption to the blood–brain barrier, a large release of neurotransmitters and an induction of neuroinflammatory mediator activity (the so-called “cytokine storm”) [13–15]. Ultimately, the disruptions from the primary injury leads to the secondary injuries [16, 17]. Secondary brain injuries rely on a cascade of molecular, biochemical and cellular events, which are triggered by the primary insult, and can involve a multitude of intertwining pathologies [18, 19]. This can include calcium or glutamate-induced excitotoxicity, oxidative stress, inflammation, neurodegeneration and dysregulated cellular metabolic pathways [20–22]. Many chronic and long-term problems can arise from the imbalance of the excitatory/inhibitory (E/I) neurotransmission that is seen after injury, with a primary example being post-traumatic epileptogenesis (PTE) [23–25]. While the initial insult itself of course cannot be medically prevented or reversed, these secondary injuries and their cascades of effects could indeed be intercepted.

Many of the most prominent aforementioned molecular cascades that are activated or enabled after a TBI are governed by kinases. In fact, phosphorylation by kinases is the most frequent and influential of all post-translational modifications (PTM) [26]. Kinases carry out phosphorylation of target proteins by the covalent binding of a phosphoryl group transferred from an adenosine triphosphate (ATP) to a serine (Ser), threonine (Thr) or Tyrosine (Tyr) residue on said protein [27]. The attachment of such a bulky, charged group can induce considerable changes in protein structure and/or function.

TBI induces an abundance of kinase triggers; cytokine release, supraphysiological intracellular calcium influx, increased neurotransmitter release and fluctuations, as well as strong inflammatory responses. The temporal post-TBI kinome is, as of now, unknown. Additional to the effects that altered kinase activity can have on cell survival and metabolism, there are also many ways in which kinases can affect ion channel function, neurotransmitter formation and neuronal receptor operations [28–35]. Despite knowing that TBI-induced kinase activity can exert this powerful influence, and with network hyperexcitability being one of the key contributors to chronic TBI pathologies, this topic has not yet been fully explored nor has it been taken as seriously as it perhaps should be in terms of therapeutic opportunity.

This project investigated the post-TBI kinome from just 4 h post-TBI to 3 days post-TBI. Wildtype mice aged between postnatal day 21 and 29 were used. We employed the controlled cortical impact (CCI) model to induce a unilateral focal brain injury in the right sensorimotor cortex. At 4 h, 24 h and 72 h post-injury, the brains were removed and the ipsilateral and contralateral hemispheres isolated. Changes in serine/threonine kinase activity were revealed using kinase assay technology whereby the ipsilateral, contralateral, and sham-operated control cortical hemispheres were compared and contrasted at each timepoint of interest after lesion induction at both the phosphopeptide level and the upstream kinase activity level. Western Blot was used to verify correlation between kinase activity with kinase expression levels in the cortex. Microelectrode Array (MEA) recordings with in vitro pharmacological interventions were used to mimic the found post-TBI kinase activity changes to gain an understanding of their influence on cortical network excitability. Finally, we harnessed the newfound functional effects of kinase activity on cortical network activity to rescue the hyperexcitability seen in the contralateral hemisphere at 24 h post-TBI. Using these techniques, we underlined and verified the overt functional relationship between TBI-induced kinase activity changes and the electrophysiological and molecular pathology that is characteristic of TBI.

## Materials & methods

### Animals & ethics statement

All animals were kept under standard 12 h day/night light conditions with constant room temperature (23 ± 2 ℃) and ad libitum access to food and water. All animal experiments were carried out in accordance with the German and European law regulations, all experimental procedures were approved by the ethics committee of the Landesuntersuchungsamt Rheinland-Pfalz (AZ 23 1777–07/G 15–1–039; 23 177–07/G20-1–112). Wildtype C57BL/6N and wildtype GAD67/GFP^(−/−)^ mice were used in this study. The number of animals was kept to a minimum, and all pursuits were taken to minimise the suffering of the mice.

### Controlled cortical impact

The controlled cortical impact (CCI) model was used to induce the unilateral lesion in the mouse right somatosensory and motor cortex. The protocol which was previously established in our lab [36] was executed. Initially, the mouse is anaesthetised with 4 Vol% isofluorane (AbbVie) until all motor reflexes have vanished. The mouse is then positioned in the stereotaxic frame whereby the anaesthesia is continued and maintained at 2 Vol% via isofluorane and oxygen ventilatory inhalation mask. For the full duration of the surgery, body temperature is maintained by a heating pad whereon the mouse is laying. Eye cream is applied to each eye as soon as the head is fixated to prevent them from drying out through the duration of the surgery (Bepanthen Augensalbe, Bayer). In the presence of a locally-applied lidocaine-containing cream (Emla salve, Aspen GmbH) an incision of median length 1 cm was made in the centre of the scalp to expose the cranial bone. With the bregma as a marking point, a 4mm^2^ cranial window was drilled over the right sensorimotor cortex, while preserving the dura matter. With careful placement of the CCI impactor tip (Impact OneTM, Leica) over the exposed area, the trauma of depth 0.8 mm was then induced. The impactor tip has a diameter of 1.5 mm and the impact was made at a velocity of 6 m/s, with contact time of 200 ms. Following impact, the exposed area was then reclosed by placing the cranial window bone back into place and sealing with cyanoacrylate-based tissue adhesive (3 M Vetbond, 3 M Animal Care Products). The scalp wound was then closed and stitched using polypropylene sutures (Ethicon). Following this, the isofluorane flow was interrupted and the anaesthesia of the animal was terminated. The animal was removed from the stereotaxic frame and returned to its cage, which remained under an infrared lamp for 1 h to aid in recovery. The control mice for this experiment underwent a sham operation; i.e., they were treated in an almost identical manner but did not have a cranial window drilled on the cranial bone or receive any mechanical impact.

### Cortex isolation & lysate formation

At each timepoint of interest post-TBI—4 h, 24 h and 72 h mice that underwent the TBI induction or the sham operation were sacrificed and their brains removed. After removal, the brain was immediately placed in a petri dish on ice and immersed in lysis buffer which consisted of Halt Phosphatase Inhibitor Cocktail (Thermo Fisher Scientific) and Halt Protease Inhibitor Cocktail EDTA free (Thermo Fisher Scientific) in dilution with N-PER Neuronal Protein Extraction Reagent (Thermo Fisher Scientific). The brain was deconstructed so that the whole ipsilateral cortical hemisphere and contralateral cortical hemisphere were carefully isolated from the remainder of the brain tissue. Whole cortical hemispheres were used due to an advanced awareness for minimum total protein concentration requirements for the kinase assay as well as other experiments which constitute this project and would be used as comparative data, e.g. Western Blots. Additionally, prospective spatial positioning of the MEA recording electrodes were taken into consideration. Sham hemispheres were labelled as ‘sham left’ or ‘sham right’ and were pooled for analysis. Lysis buffer was added to each hemisphere and lysis was promoted by the gentle pipetting of the mixture. After 30 min, the lysing cortices were placed on the rotary wheel in the cold room to incubate for 30 min at 4 ℃. The vials were then centrifuged at maximum speed at 4 ℃ for 15 min and the supernatant was collected. Protein quantification was achieved using the Pierce BSA Protein Assay Kit (Thermo Fisher Scientific). For the kinase assay experiments, lysates were aliquoted and diluted in accordance with the amount of protein needed per assay array; serine-threonine kinase (STK) arrays requiring 1 μg of protein per array. Lysates were aliquoted to avoid freeze–thaw cycles and stored at − 80 ℃ until use. For Western Blot experiments, lysates were prepared for blotting with 4 × LDS (NuPage LDS Sample Buffer, Invitrogen) and dithiothreitol (DTT) (Thermo Fisher Scientific) to an end protein concentration of 1.5 μg/μL.

### Kinase assay

In order to find out which kinases were more active or less active in the post-TBI brain in comparison to the sham control cortical hemispheres, we used the PamGene^®^ PamChips^®^ for kinase assay experiments. This protocol uses a flow-through microarray assay to determine the activity of serine/threonine or tyrosine kinases. Phosphorylation activity is first determined using immobilised peptide substrates on the chip arrays with 144 peptides on the STK array. The concept is that the kinases present in the cortical brain lysates will phosphorylate their target peptide substrates which are immobilized on each array. Lysates were created as described above from the ipsilateral, contralateral and sham-operated hemispheres of the experimental mice for each timepoint of interest post-TBI. On the day of experimentation, an aliquot from each lysate sample (TBI ipsilateral n = 3, TBI contralateral n = 3, sham n = 4, for each timepoint) was thawed and kept on ice. Following a blocking and washing step of the array chips (3 chips per run, 4 arrays per chip), fresh sample mixes were made and combined with an STK primary antibody for tagging. These sample mixes are pipetted onto the delicate arrays. Following this, the STK array requires an additional detection mix which includes an STK FITC-labelled antibody. The phosphorylation activity is then detected by these fluorescently labelled antibodies and recorded by a CCD-camera at different exposure durations and intervals in the PamStation^®^. The data workflow consisting of image quantification, quality control, statistical analysis and visualisation is performed using the BioNavigator^®^ software. From these, two important things can be analysed. First, which of the peptide substrates were more phosphorylated or less phosphorylated by the increased activity or decreased activity of the kinases present in the post-TBI cortical brain lysates. This can be determined for each peptide by its fluorescent signal strength and its respective log fold change (LFC). The peptide difference statistic is calculated for each peptide comparing two conditions using the log_2_ kinase activity profiles measured on the chips. The peptide difference statistic is the Signal to Noise Ratio (SNR) of the difference between the log_2_ kinase activity profiles.

$$p= \frac{\overline{{x }_{T}}- \overline{{x }_{c=0}}}{\sqrt{{\sigma }_{T}^{2}+{\sigma }_{c}^{2}}}$$

where *p* = peptide difference statistic, *x̅* = mean log_2_ kinase activity of *T* (Test) or *C* (Control) conditions, √σ2 = standard deviation of kinase activity of the two conditions.

Secondly, the differential kinase activity in the post-TBI cortices compared to the sham-operated cortices. This is predicted using the Upstream Kinase Analysis (UKA) algorithm which uses the publicly accessible database that documents kinase-substrate relationships. Sham cortices could be pooled as no statistical difference was found between ‘sham left’ and ‘sham right’ cortical hemispheres at any timepoint post-TBI (Supplementary Fig. 7).

The UKA returns the following scores: Kinase Statistic (represents the change in kinase activity, < 0 = inhibition, > 0 = activation), Specificity Score (represents the specificity of the change in kinase activity; a higher specificity score means a lower chance that the observed effect could have been obtained by a random set of peptides) and the Kinase Score (the sum of the significance and the specificity).

$$s=b*\left({U}^{T}p\right)$$

$${b}_{i}=1/{a}_{i}$$

where* s*: normalized kinase statistic is the weighted average of *p*, the peptide difference statistic. *b* is a normalization vector, *ai* is the number of nonzero elements in column i of *U* (number of peptides belonging to a kinase).

Six databases were queried for kinases that are verified and/or predicted to phosphorylate these sites. This results in a binarized peptide-to-kinase matrix, *U*. To assess whether the normalized kinase statistic is significant (significance score), the samples of the kinase activity profiles are permuted M times. Then the normalized kinase statistic is calculated for the permuted values. The significance score is calculated using the number of times the newly calculated normalized kinase statistic is higher than the original normalized kinase statistic. The specificity score is calculated in the same way as the significance score but on the basis of random permutations of the peptides. A higher specificity score means a lower chance that the observed effect could have been obtained by a random set of peptides. The final score is the sum of the significance score and the specificity score. The final score is used to rank the included kinases for putative involvement in the observed experimental differences.

### Western blots

Quantitative protein detection was used in order to define the expression of target proteins and/or kinases in the cortical brain lysate samples that were created as described above. These lysates were created at each timepoint of interest post-TBI; 4 h, 24 h and 72 h, in order to track the change in target expression at the protein level over time proceeding induction of the cortical injury. Sham, post-TBI ipsilateral and post-TBI contralateral hemispheres (n = 3–8 for each) were analysed. 8–12% Bis–Tris Acetate gels (Thermo Fisher Scientific) were used to run the samples and they were transferred to a polyvinylidene difluoride (PVDF) membrane (Carl Roth, CA, USA) which had previously been activated with methanol and blocked for 30 min with 4% non-fat dry milk in tris-buffered saline with Tween (TBST) (Sigma-Aldrich). As a loading control for target antibodies, goat anti-GAPDH (1:1,000; A303-878A) was used along with the secondary antibody donkey anti-goat (1:10,000; Dianova). The primary antibody was rabbit anti-p44/42 (Erk1/2) (1:2,000; Cell Signalling Technology) and was incubated overnight at 4 °C. For detection, a secondary horseradish peroxidase (HRP)-conjugated goat anti-rabbit (1:10,000; Dianova) antibody was used for 30 min at room temperature. The membrane was imaged using electrochemiluminescence (ECL) detection solution (Merck Millipore) and signals were acquired with Image Studio Lite 5.2 (Bio-Rad, Hercules). Detected signals were normalized to the signal of the loading control and standardised to the signal obtained from lysates originating from the cortices of the sham-operated littermates. Sham hemispheres were verified as having no statistical difference between ‘sham left’ and ‘sham right’ for both ERK1 and ERK2 and at every analysed timepoint of interest post-TBI (Supplementary Fig. 7). Three of the phosphopeptides that showed a significant over-phosphorylation on the kinase assay array at 24 h post-TBI were also tested via Western Blot for their phosho-protein/total protein ratio. Primary antibodies rabbit anti-phospho-tyrosine hydroxylase (1:1,000, Cell Signalling Technology), rabbit anti-tyrosine hydroxylase (1:1,000, Cell Signalling Technology), rabbit anti-phospho-Ca_v_1.2 (1:1,000, Thermo Fisher Scientific), rabbit anti-Ca_v_1.2 (1:1,000, Synaptic Systems GmbH), rabbit anti-phospho-CFTR (1:1,000, Thermo Fisher Scientific), mouse anti-CFTR (1:500, Thermo Fisher Scientific) were used with either goat anti-rabbit (1:10,000; Dianova) or goat anti-mouse (1:10,000, Dianova) HRP-conjugated secondary antibody.

### Microelectrode array (MEA)

MEA experiments consisted first of specific pharmacological kinase inhibition/activation to mimic the post-TBI kinase activity changes that were unveiled through the kinase assay, and then subsequent experiments using kinase inhibition to rescue the network hyperexcitability of the contralateral hemisphere. For the pharmacological mimicking experiments, wildtype mice were anaesthetised with 4 Vol% isoflurane and then decapitated. The brain was immediately removed and placed in ice-cold artificial cerebrospinal fluid (ACSF), oxygenated with 95% O_2_ and 5% CO_2_, consisting of (in mM): NaCl (125), KCl (3), MgSO4 × 7H2O (1.3), CaCl2 × H2O (2.5), NaHCO3 (26) and d-glucose (13) and at a pH of 7.4. Brains were then cut into coronal sections of 350 μm thickness using a microtome (VT1200 S, Leica) for microelectrode array (MEA) recordings. The cortical slices of interest were collected in oxygenated ACSF and incubated at room temperature for at least 40 min prior to recordings. Brain slices that were to be treated with the pharmacological inhibitors or activators underwent varying bath application and/or incubation times. Slices being treated with the ERK inhibitor FR180204 (Tocris Bioscience) were incubated in ACSF + 25 μM FR180204 for at least 1 h prior to recordings. Slices being treated with the PKC-activating phorbol 12-myristate 13-acetate (PMA) (Tocris Bioscience) at a 1 μM concentration and PKC-inhibiting chelerythrine chloride (Tocris Bioscience) at a 5 μM concentration did so in a wash-in fashion for 15 and 25 min, respectively, due to their fast effect time. All pharmacological kinase manipulator drugs were dissolved in dimethyl sulfoxide (DMSO) and this was controlled for with supplementary recordings showing no difference between ACSF control recordings versus ACSF + DMSO recordings (Supplementary Fig. 1B). Extracellular recordings of the spontaneous neuronal activity and amplitude responses from an input/output curve protocol were acquired in the acute cortical brain slices with a two- chamber MEA system (MEA2100 System, Multi Channel Systems MCS GmbH). Each MEA chip had 60 electrodes (60MEA200/30iR; Multi Channel Systems MCS GmbH) with a diameter of 30 μm each arranged with a distance of 200 μm between each electrode. The acute cortical slices with a thickness of 350 μm were then placed on the MEA, with the outer cortical border (correlating to cortical layer 1) aligned along the first row of electrodes. Electrode rows number 2, 3 and 4 corresponded with cortical layers 2–4, respectively, and were used for this analysis of neuronal activity. Slices were stabilised on the MEA with a platinum grid and were constantly perfused at 32 ℃ with heated and oxygenated ACSF which contained (in mM) NaCl (125), KCl (3), MgSO4 × 7H2O (1.3), CaCl2 × H2O (2.5), NaH2PO4 × H2O (1.25), NaHCO3 (26) and d-glucose (13). Initially, the slices were left to rest on the MEA chip and were monitored for at least 30 min before any recordings began to ensure stable contact of the slice with the electrodes. Spontaneous events were recorded with the Multi Channel Experimenter 2.2 (Multi Channel Systems MCS GmbH) using a 50 kHz sampling rate and a Butterworth highpass 2.0 Order Filter with 200 Hz cut-off. Events larger than the five-fold standard deviation of the noise were collected in five-minute traces using the Multi Channel Analyzer 2.2 (Multi Channel Systems MCS GmbH). The input/output curve protocol consisted of input stimulation starting at 500 mV and increasing in steps of 500 mV until a maximum of stimulation of 5000 mV. The duration between each voltage input stimulation was 40 s, with a total of 3 cycles of this protocol being carried out per slice. The Multi Channel Analyzer 2.2 recorded the peak value of each resultant local field potential (LFP) amplitude that was an output from the 500–5000 mV inputs. For each input, the mean average output was obtained. For the wash-in experiments with PMA and chelerythrine, a 2500 mV stimulation was issued once every 60 s. A 10-min baseline recording was initially obtained before the wash-in of either PMA or chelerythrine. The 2500 mV stimulations then continued once every 60 s for either 15 or 25 min, respectively. All extracellular voltage outputs LFPs were normalised to the baseline recording.

For the 24-h post-TBI MEA experiments, wildtype mice which had undergone either a CCI or sham surgery 24 h previously were anaesthetised with 4 Vol% isoflurane and then decapitated. Brain removal, sectioning, ACSF, slice storage and MEA placement was carried out in a way that is identical to what is described above. Spontaneous events were recorded with the Multi Channel Experimenter 2.2 (Multi Channel Systems MCS GmbH) using a 50 kHz sampling rate and a Butterworth highpass 2.0 Order Filter with 200 Hz cut-off. Events larger than the five-fold standard deviation of the noise were collected in five-minute traces using the Multi Channel Analyzer 2.2 (Multi Channel Systems MCS GmbH). The first five-minute trace for each post-TBI hemisphere (ipsilateral n = 12, contralateral n = 13) was recorded before the wash-in of chelerythrine. Another five-minute trace was recorded for each hemisphere after the 25-min chelerythrine wash-in duration. Sham slices (n = 17) did not undergo a wash-in of chelerythrine and were used as controls for the post-TBI hemispheres as well as for any spontaneous event frequency changes that may naturally occur during the total recording period on the MEA chip.

### Immunofluorescent staining & confocal microscopy

Two 24-h post-TBI brains were removed and left in 4% paraformaldehyde at 4 ℃ for 24 h and rinsed in phosphate buffer (PB) 0.1 M. Brains were placed in 30% sucrose in 0.1 M PB for 24 h and sectioned into 30 mm on a freezing microtome (model CM 1325; Leica Microsystems). For free floating staining, coronal subslices were washed with phosphate buffered saline (PBS) 0.01 M and then blocked and permeabilized for two hours at room temperature (RT) with a solution containing 7% normal donkey serum (017–000–121, Dianova) and 0.8% Triton X-100 in 0.01 M PBS. The sections were incubated for 3 days at 4 ℃ in the primary antibodies rabbit recombinant monoclonal anti-PKC alpha [Y124] (1:100, ab32376, abcam) or rabbit recombinant monoclonal anti-PKC gamma [EPR28643-68] (1:500, ab317315, abcam), polyclonal chicken anti-GFAP (1:500, 173 006 Synaptic Systems) and polyclonal guinea pig anti-NeuN (1: 2000, 266 014, Synaptic Systems) in a staining buffer containing 2% bovine serum albumin (001–000–161, Jackson ImmunoResearch, Dianova) with 0.05% azide and 0.3% Triton X-100 in 0.01 M PBS. After washing in 0.01 M PBS, slices were incubated in the secondary antibodies and 0.5 μg/mL DAPI (A4099.0005, AppliChem) for two hours at RT in a staining solution containing 2% bovine serum albumin (001–000–161, Dianova) with 0.05% azide. Secondary antibodies were Alexa Fluor^®^ 647-conjugated and raised against rabbit IgG (Donkey Anti-Rabbit IgG (H + L), 1:200, 711–605-152, Jackson ImmunoResearch) and Alexa Fluor^®^ 488-conjugated and raised against chicken (Donkey Anti-Chicken IgG (H + L), 1:200, 703–545-155, Jackson ImmunoResearch, Dianova) and Cy3-conjugated and raised against guinea pig (Donkey Anti-Guinea Pig IgG (H + L), 1:400, 706–165-148, Jackson ImmunoResarch, Dianova). After washing in 0.01 M PBS, slices were mounted onto Superfrost™ Plus Adhesion microscope slides and coverslipped with Fluoromount-G (SouthernBiotech) mounting medium. Images were acquired using the Visitron Spinning Disc confocal microscope with an ablation laser on a Nikon stand with a CSU-W1 spinning disk (25 µm and 50 µm pinholes) from Yokogawa. Objective was at 40× (1.25 NA, water).

### Statistics

The data was statistically evaluated and analysed with use of PamGene BioNavigator or with GraphPad Prism 10 software. Results are shown as mean ± standard error of the mean, unless otherwise stated. Two-way ANOVA with post-hoc Šídák’s multiple comparisons test was used for the MEA input/output curve analyses. Unpaired t-test was used for the mean spike frequency spontaneous activity analysis for the ERK-inhibition MEA experiments. Paired t-test was used for the PMA and chelerythrine wash-in MEA experiments. Paired t-test was used for the 24-h post-TBI spontaneous activity MEA analysis for before and after chelerythrine wash-in within each hemisphere group (ipsilateral, contralateral and sham), and ordinary one-way ANOVA with post-hoc Tukey’s multiple comparisons test for intergroup differences between ipsilateral, contralateral and sham hemispheres at the timepoint before and the timepoint after chelerythrine addition. Ordinary one-way ANOVA with post-hoc Tukey’s multiple comparisons test was used for quantitative Western Blot analysis. Significant differences between experimental groups are indicated by asterisk ^*^P < 0.05; ^**^P < 0.01; ^***^P < 0.001.

## Results

### TBI induction using CCI model & establishing timepoint of interest post-injury

**Fig. 1:**
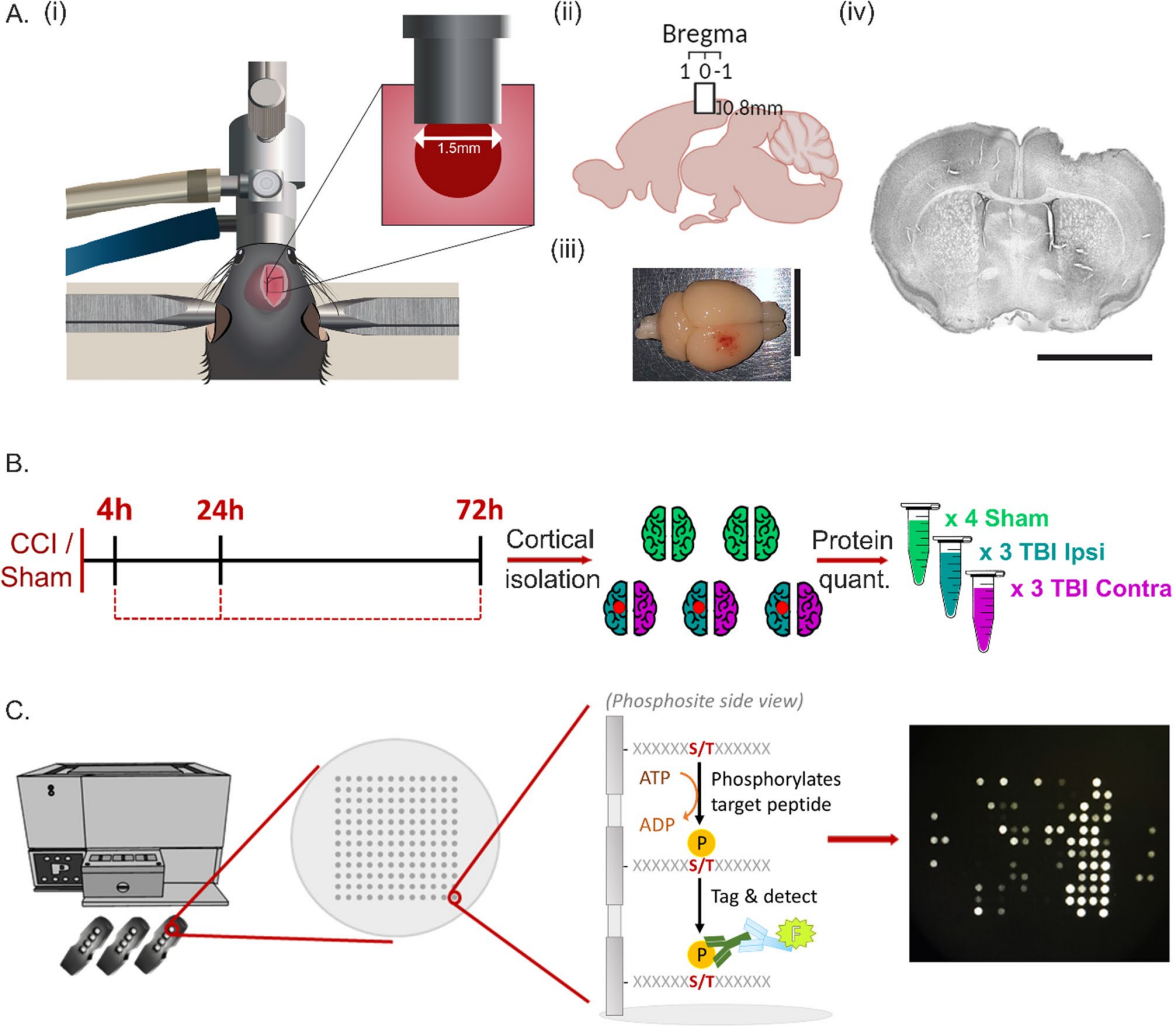
Experimental design: CCI Induction, Cortical Isolation & Kinase Assay Protocol at 4 h, 24 h and 72 h post-TBI. **A** (i) Demonstrative illustration of the CCI setup including stereotaxic frame fixation and ventilation of oxygen and anaesthesia via an inhalation mask. The mouse lay on a heated pad throughout the surgery to maintain body temperature. Unilateral impact was made through a 4mm2 cranial window (indicated by square) on the right sensorimotor cortex. The impactor tip had a diameter of 1.5 mm. (ii) The impact was made at Bregma 0, with a depth of 0.8 mm. (iii) Representative PFA-perfused mouse brain showing the injury lesion directly after a TBI. Scale bar = 10 mm. (iv) Representative Nissl-stained coronal slice of a TBI-treated animal. **B** Experimental protocol for the preparation of the sample lysates for the kinase assay. At 4 h, 24 h and 72 h post-TBI/sham operation, the animals were sacrificed and the brain removed. The ipsilateral (n = 3 per timepoint), contralateral (n = 3 per timepoint) and sham-operated (n = 4 per timepoint) cortical hemispheres were isolated from the brain. Individual sample protein quantification was executed and aliquots created. **C** The kinase assay samples with active kinases are applied to the chip arrays, which then phosphorylate downstream target peptide substrates in accordance with which kinases are active. The transferred phosphate group is then fluorescently tagged and imaged by a CCD-camera at different exposure durations and intervals

Unilateral TBI was induced in anaesthetised mice with the controlled cortical impact (CCI) model as previously established in our lab (36) and in full accordance with local and EU-ethics regulations. Timepoints of interest were based on a combination of realistic therapeutic opportunity for TBI patients on presentation to a hospital or clinic, times deemed most interesting in terms of molecular cascades, and effects and post-TBI timepoints previously examined in our laboratory. At 4 h, 24 h and 72 h post-injury, cortical hemisphere homogenate lysates were prepared for kinase assay and upstream kinase activity was sufficient to produce mensurable fluorescent signals on the phosphopeptide array (Fig. 1).

### Hemispheric kinase activity begins with high synchronicity at 4 h post-TBI but diverges by 72 h post-injury

#### 4 Hours post-TBI

**Fig. 2:**
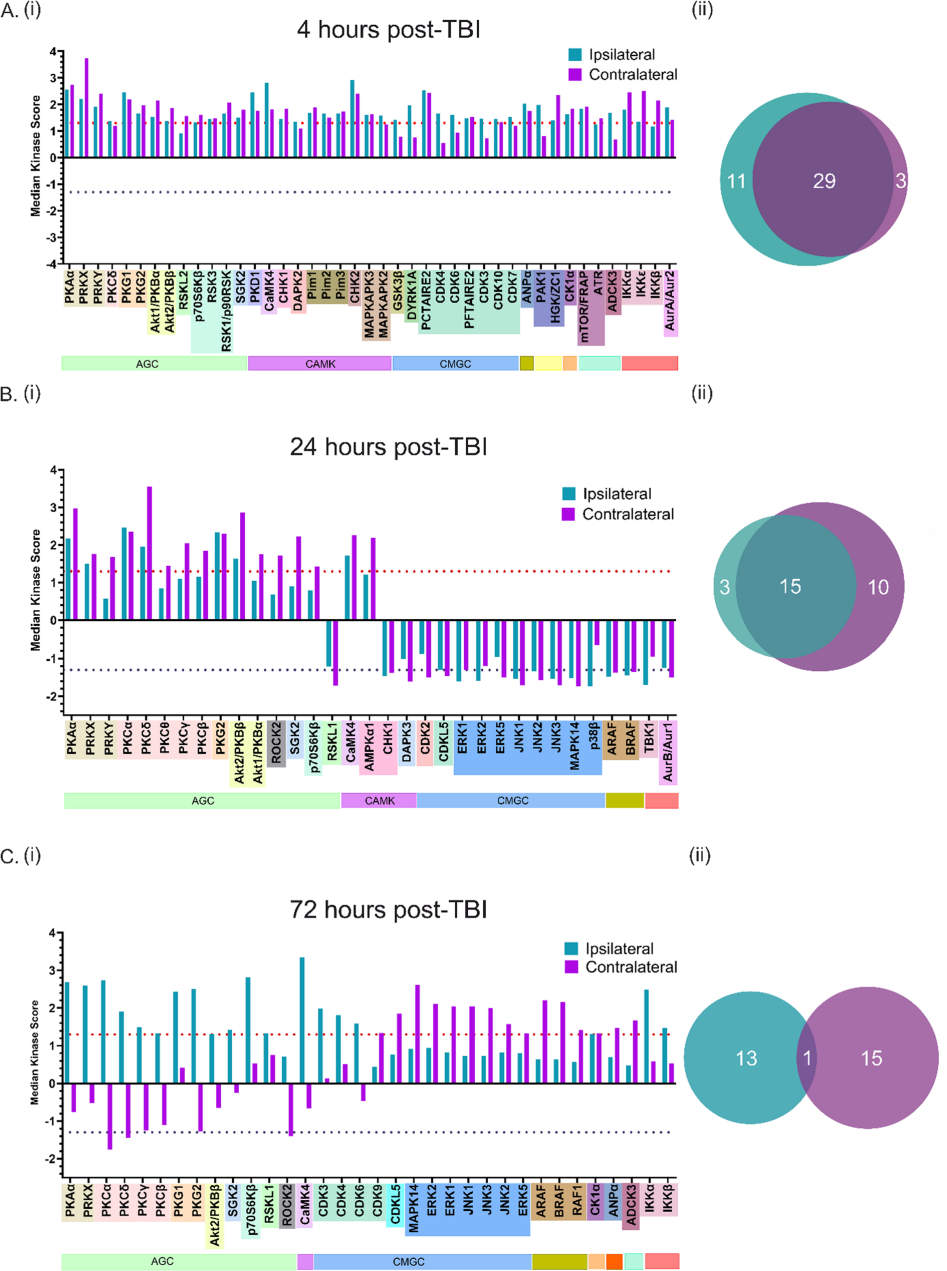
Kinase Activity is Dynamic from 4 to 72 h post-TBI, with Interhemispheric Kinome Synchronicity Reducing over Time. Median kinase scores which surpassed the threshold for significant over- or under- activity in either the ipsilateral or contralateral hemisphere relative to sham hemispheres is shown for each timepoint. All kinase activity is presented relative to sham. Kinases that belong in the same kinase group are indicated with the kinase group name underneath, e.g. AGC. All kinases that belong to the same kinase family within a kinase group are coloured with the same textbox colour. Threshold for differentially active kinases = – log10(0.05) = ( ±)1.3. For graphs **A** (i), **B** (i) and **C** (i) the threshold for significant overactivity is depicted by the dotted red line and that of underactivity by the dotted purple line. p = 0.05. **A** (ii), **B** (ii) and **C** (ii) Venn diagrams visualising the proportion of differentially active kinases that surpassed the threshold in either the ipsilateral (blue) hemisphere only, the contralateral (purple) hemisphere only, or in both hemispheres, at each timepoint. All individual kinase name abbreviations can be found in Supplementary Table 1

All upstream serine/threonine kinase (STK) activity changes at 4 h post-injury show an increase. The median kinase score graphs (Fig. 2) present any kinase that was significantly changed (kinase score passed the threshold of ± 1.3) in either one or both hemispheres relative to sham-operated control hemispheres at the same timepoint. The high synchronicity between hemispheres at this acute timepoint after injury is apparent from the median kinase score graph (Fig. 2A, i), with the overlap in kinase activity between ipsilateral and contralateral hemisphere coming to 67.4% (Fig. 2A, ii) despite the obvious phenotypic differences. The AGC kinase group (indicated with pale green box)—named after its protein kinase A (PKA), cGMP-dependent protein kinase (PKG) and protein kinase C (PKC) members—accounts for the largest proportion of active kinases at this timepoint. Upstream PKA activators include chemokines such as interleukin 8 (IL-8) and chemokine ligand 2 (CCL-2), as well as neurotransmitters such as norepinephrine, glutamate, dopamine and acetylcholine. PKG1 and PKG2 are activated downstream of nitic oxide (NO); increased NO levels being classically associated with post-TBI pathogeneses such as glutamate excitotoxicity and mitochondrial dysfunction. In the Ca^2+^/calmodulin-dependent protein kinase (CAMK) group (indicated with pale purple box), which are activated by increases in intracellular calcium ion concentrations, nine out of ten of the significantly active CAMKs are increased in activity in both the ipsilateral and the contralateral hemisphere; an insight to the global effect that the initial unilateral cortical injury has. The fact that so many of the CAMK group are active reflects the calcium-centric nature of the malfunctions that are occurring at this early timepoint proceeding the primary injury. The one CAMK that passes the threshold for increased activity exclusively in the ipsilateral hemisphere is the death-associated protein kinase 2 (DAPK2); a positive regulator for programmed cell death (PCD). The CMGC family also have a large representation at 4 h post-TBI. Interestingly, the most abundant CMGC family (indicated with pale blue box) activity at this timepoint are the cell cycle cyclin dependant kinases (CDKs), rather than the growth- and stress- response mitogen-activated protein kinases (MAPKs), which instead have a stronger influence at later timepoints. The strong pro-apoptotic effects of cell cycle activation by CDKs in post-mitotic neurons may contribute to the neurodegeneration that occurs by virtue of brain injury. The acute inflammatory response is shown by the overactivation of IkappaB kinase (IKK) complex subunits; being crucial upstream players in the nuclear factor kappa-light-chain-enhancer of activated B cells (NFkB) signalling pathway which is fundamental for immunoregulation.

#### 24 hours post-TBI

By 24 h post-TBI, some kinase families make a radical switch from being significantly overactive to significantly underactive. This kinase activity change ensues in both hemispheres in a way that is still synchronous, as can be seen in Fig. 2B. The degree of hemispheric kinase activity overlap remains high, although now slightly decreased in comparison with the 4-h post-TBI timepoint. The AGC kinase group still comprises a large portion of the past-threshold kinases at this timepoint. This time, the PKC family emerges and has the largest claim compared to the other group members, where it had one of the smallest influences at the previous timepoint. Interestingly, in almost all cases, the overactivity of PKC is either exclusive to or more prevalent in the contralateral hemisphere. At 24 h, there are only four past-threshold CAMK members in one or both hemispheres, with half of those actually being significantly reduced in activity relative to sham. This change may reflect the evolving pathology of the injury from a calcium-centric initiation to a now more colourful development of the damage as it advances from primary to secondary injuries. This is also apparent when we note that the past-threshold CAMKs at this timepoint are even behaving in a way that is beneficial to the neurons. Death-associated protein kinase 3 (DAPK3), for example, is significantly decreased in activity in the contralateral hemisphere. This shows first a dramatic change from the 4-h overactivity of fellow DAPK member DAPK2, and second, neuroprotective measures that are exclusive to the contralateral hemisphere. This is clear too with the 5’-andenosine-monophosphate-activated protein kinase (AMPK) alpha 1 (AMPKα1) catalytic subunit, which is activated in response to severe cellular energy depletion and helps to reinstate a healthy AMP/ATP ratio. Although displaying a clear trend towards overactivity in the ipsilateral hemisphere, it is past-threshold for differential kinase activity in the contralateral hemisphere only. In the CMGC family, there is now exclusively a reduction in activity amongst all past-threshold candidates. A much lesser role is being played by the CDKs at the 24-h timepoint, replaced instead by a very strong MAPK influence. The MAPK group activity at this timepoint is interesting for several reasons. The pro-survival extracellularly-regulated-kinase (ERK) 1/2 (ERK1/2) and pro-apoptotic c-Jun-N-terminal kinases (JNK) MAPK members have a concurring change in activity in the same direction and at the same time. It is intriguing that both pathways, which have opposing outcomes, undergo these changes simultaneously. This may infer that these kinases carry a role other than that in cell death or survival in the post-injury brain.

#### 72 hours post-TBI

At the 72-h post-TBI timepoint, a dichotomous kinase behaviour between the ipsilateral and contralateral hemispheres is evident. This is interpretable from the Median Kinase Score graph in Fig. 2C, i, but also from the fact that there is now only one kinase—casein kinase 1 alpha (CK1α)—that has an above-threshold activity in both hemispheres, as depicted in Fig. 2C, ii. Where the AGC kinases remain strongly overactive in the ipsilateral hemisphere, they are either irrelevant or significantly underactive in the contralateral hemisphere. It is interesting to consider the changes occurring within the AGC group between the 4-h and the 72-h timepoint. PKAα (the catalytic subunit of PKA) and PRKX, members of the PKA family, are highly active at this timepoint and have maintained an above-threshold activity in the ipsilateral hemisphere since the 4-h post-injury timepoint. Since upstream activators, e.g. chemokines, are present in lesioned tissue from injury onset, this is quite logical. This also goes for the excitotoxic indiscriminate excitatory neurotransmitter release upon brain impact, which also activates PKA kinases [37]. The more intriguing AGC activity lies in the PKC family. At 24 h after injury, activity in the PKC family dramatically emerged and its members either exclusively overactive in, or more strongly active in, the contralateral hemisphere. At 72-h post-TBI, the PKCs are noticeably the most underactive AGC kinase family for the contralateral hemisphere, while PKC activity in the ipsilateral hemisphere soars (Fig. 2C, i). PKCα is the family member showcasing the highest activity in the ipsilateral hemisphere at this time, which is activated mostly by calcium, but also by hypoxia and mechanical strain [38]. For the CMGC kinase group, we note that the CDK activity has reverted back to the increased level observed at the 4-h timepoint in the ipsilateral hemisphere. This may reflect a wave of inflammation which is highly CDK-associated, such as the activation and accumulation of residential microglia and astrocytes, in the lesioned tissue. It may also be highly apoptotic in nature, since noxious cell cycle progression in post-mitotic brain tissue is detrimental. The contralateral hemisphere sees no such biphasic CDK activation, instead having been affected by the initial injury at the very acute phase which faded away over time. The upstream NFkB activator complex, IKK, is also once again overactive at 72 h post-TBI and only in the ipsilateral hemisphere, which further supports this idea. Instead, the contralateral hemisphere displays a striking MAPK overactivation, while the ipsilateral hemisphere shows no past-threshold MAPK activity. Once again, the pro-survival ERKs and the pro-apoptotic JNKs share the same rate of activity, yet undergo huge activity fluctuations from one timepoint to another. This perhaps suggests that the MAPK family may have additional functions post-TBI, in addition to cell death or survival through its (in)activation.

We found the kinase changes occurring at the 24-h post-TBI timepoint to be the most compelling. This is the timepoint at which pro-survival ERK/MAPK activity transiently and strongly decreases; seemingly inappropriate considering it is just one day after injury. It is also this timepoint that the PKC family emerges from previously subthreshold activity, with a particular strength in the contralateral hemisphere. To explore the consequences of the dynamic kinase activity profile of ERK1/2 and PKC in terms of their effect on cortical network excitability, we next utilised pharmacological intervention alongside extracellular electrophysiological MEA recordings using in vitro mouse coronal brain slices.

### ERK expression & activity fluctuate after TBI and its inhibition has a significant effect on cortical network excitability

**Fig. 3:**
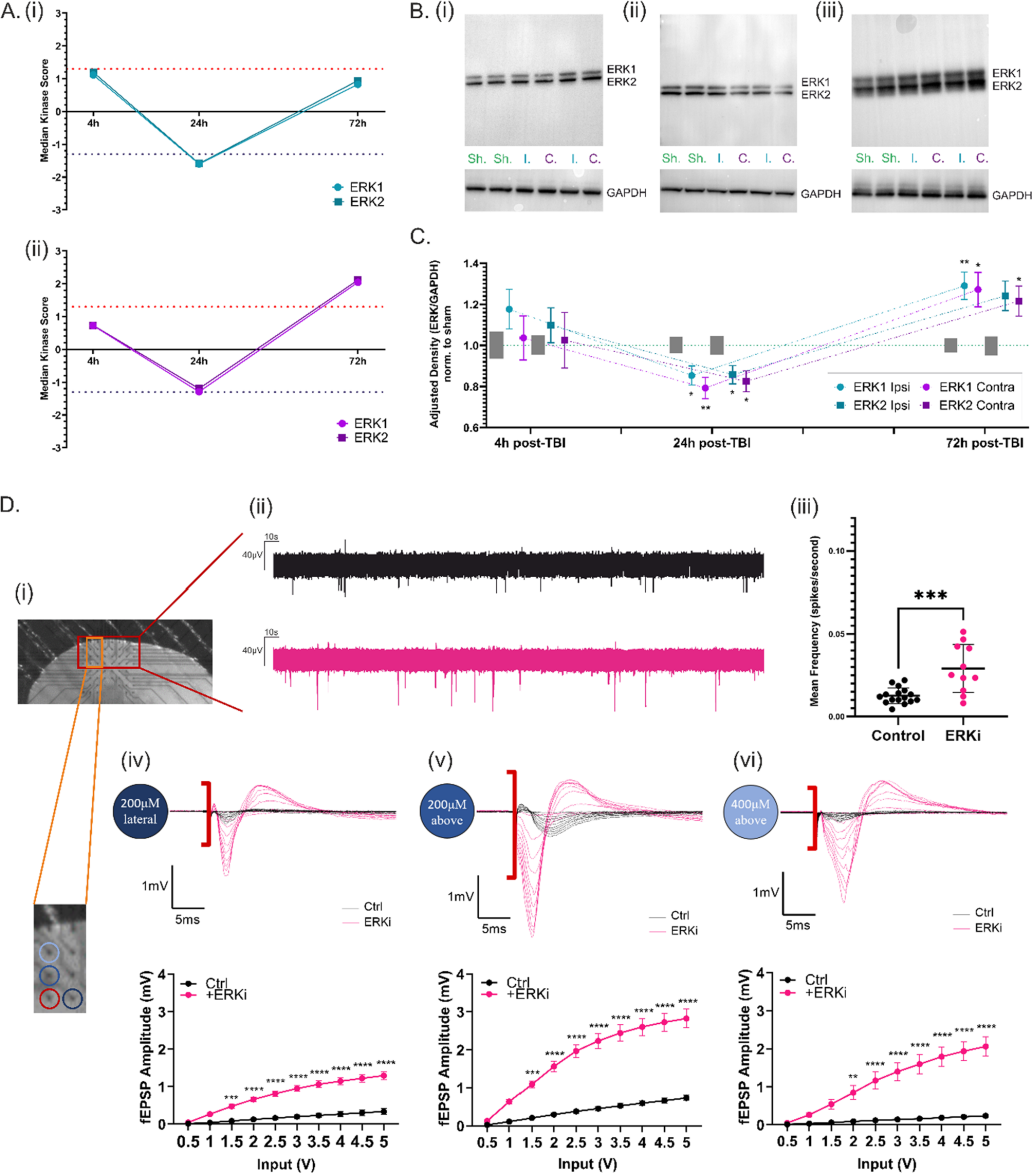
Pharmacologically Mimicking the Reduced ERK1/2 Kinase Activity and Protein Expression found at 24 h post-TBI has a Significant Influence on Spontaneous and Evoked Cortical Excitability. **A** (i) Temporal profile of ERK1/2 activity from 4 to 72 h post-TBI in the ipsilateral cortical hemisphere. (ii) Temporal profile of ERK1/2 activity from 4 to 72 h post-TBI in the contralateral cortical hemisphere. **B** Representative blots for cortical ERK1/2 expression from (i) 4 h, (ii) 24 h and (iii) 72 h post-TBI, with GAPDH housekeeper protein for normalisation. **C** Temporal profile of the fluctuating ERK1/2 protein expression relative to sham from 4 to 72 h post-TBI showing a correlation of ERK1/2 expression with its respective activity at matching timepoints. Grey bars show the standard error of mean of the sham hemispheres (n = 6–18). **D**. (i) Exemplary placement of the coronal ex vivo brain slice on the MEA chip with the recording electrodes correlating to cortical layers 1–4 outlined in red. (ii) Representative five-minute traces of the spontaneous spiking activity from the control group (black) and the ERK-inhibited group (pink). (iii) Analysis of the spontaneous activity via mean spiking frequency between the control group (black) and the ERK-inhibited group (pink). (iv) Overlaid representative LFPs of control (black) and ERK-inhibited (pink) slices from 200 μV lateral to the stimulation (0.5–5 V) electrode. Below, the correlating Input/Output Curve analysis from all recordings. (v) Overlaid representative LFPs of control (black) and ERK-inhibited (pink) slices from 200 μV above the stimulation (0.5–5 V) electrode in the direction of the dorsal cortical surface. Below, the correlating Input/Output Curve analysis from all recordings. (vi) Overlaid representative LFPs of control (black) and ERK-inhibited (pink) slices from 400 μV above the stimulation (0.5–5 V) electrode in the direction of the dorsal cortical surface. Below, the correlating Input/Output Curve analysis from all recordings. n = 11 (control) and n = 14 (ERKi). Statistical analysis for Western Blot was carried out using one-way ANOVA with post hoc Tukey’s multiple comparison test. Statistical analysis for mean spike frequency was carried out using unpaired t-test. Statistical analysis for input–output curves was carried out using two-way ANOVA with post hoc Šídák’s multiple comparisons test. p < 0.05

Now that this compelling ERK1/2 temporal kinase activity profile (Fig. 3A) for the post-TBI ipsilateral (i) and contralateral (ii) cortical hemispheres has been uncovered, we were interested to also ascertain the protein expression levels of ERK1/2 from 4 to 72 h post-TBI. Figure 3C displays the quantitative Western Blot analysis, with a slightly increased but not significantly changed ERK1/2 expression at 4 h post-injury, a significant decrease in ERK1/2 expression at 24 h post-injury, followed by a significant increase in ERK1/2 expression at 72 h post-injury (with the exception of ERK2 in the contralateral hemisphere). The expression levels of ERK1/2 at the protein level strongly mirror the kinase activity levels of ERK1/2 at the same timepoints, revealing a direct correlation between ERK1/2 expression levels and ERK1/2 activity levels in the cortex. Western Blots from cortical lysates collected at 4 h, 24 h and 72 h post-TBI are shown in Fig. 3B.

We also uncovered a further significant decrease in ERK1/2 expression at one-week post-TBI (Supplementary Fig. 4C), showing that ERK expression and/or activity continues to fluctuate even past the 3-day point post-TBI.

A selective activator for ERK1/2 unfortunately does not currently exist. However, a specific ERK1/2 inhibitor—FR180204—was used in the electrophysiological MEA recordings which pharmacologically mimicked the ERK1/2 underactivity and significant downregulation that is seen at 24 h post-TBI. Coronal slices were placed on a 62-electrode MEA chip with the first row of electrodes consistently aligned with cortical layer 1, as shown in Fig. 3D, i. Inhibiting ERK1/2 using 25 µM of FR180204 caused an increase in spontaneous activity of the cortex as shown by the activity trace and the mean spike frequency analysis of control versus ERK-inhibited slices in Fig. 3D, (ii) and (iii), respectfully. An examination of the evoked activity changes between control and ERK-inhibited slices by way of an input–output curve analysis was also carried out. A stimulation series from 0.5 V to 5 V with 0.5 V-step incrementations was executed in Layer IV of the cortex with evoked fEPSP output amplitudes being recorded at 200 µm lateral to, 200 µm above and 400 µm above the stimulation electrode. At 200 µm lateral to and 200 µm above the stimulation electrode, the ERK-inhibited slices showed a significantly increased evoked fEPSP amplitude from all stimulation inputs from 1.5 to 5 V, with representative traces also shown, in Fig. 3D, iv, v. Even at the distance of 400 µm above the stimulation electrode, the ERK inhibition caused a significant increase in evoked fEPSP amplitudes for all stimulations from 2 to 5 V, again shown alongside representative traces in Fig. 3D, vi. This dramatic effect of ERK inhibition on the electrophysiological activity of the cortex was unprecedented. To ensure these effects were indeed mediated by the inhibition of ERK, further MEA experiments with the pharmacological inhibition of ERK’s sole upstream activator, mitogen-activated protein kinase kinase (MEK), were carried out (Supplementary Fig. 1A). These experiments again displayed a significant increase in evoked fEPSP amplitudes, validating that inhibition of the ERK/MAPK pathway does in fact cause a hyperexcitation of the cortical network.

### PKC activity undergoes dynamic changes after TBI and its activation or inhibition has a significant effect on cortical network excitability

**Fig. 4:**
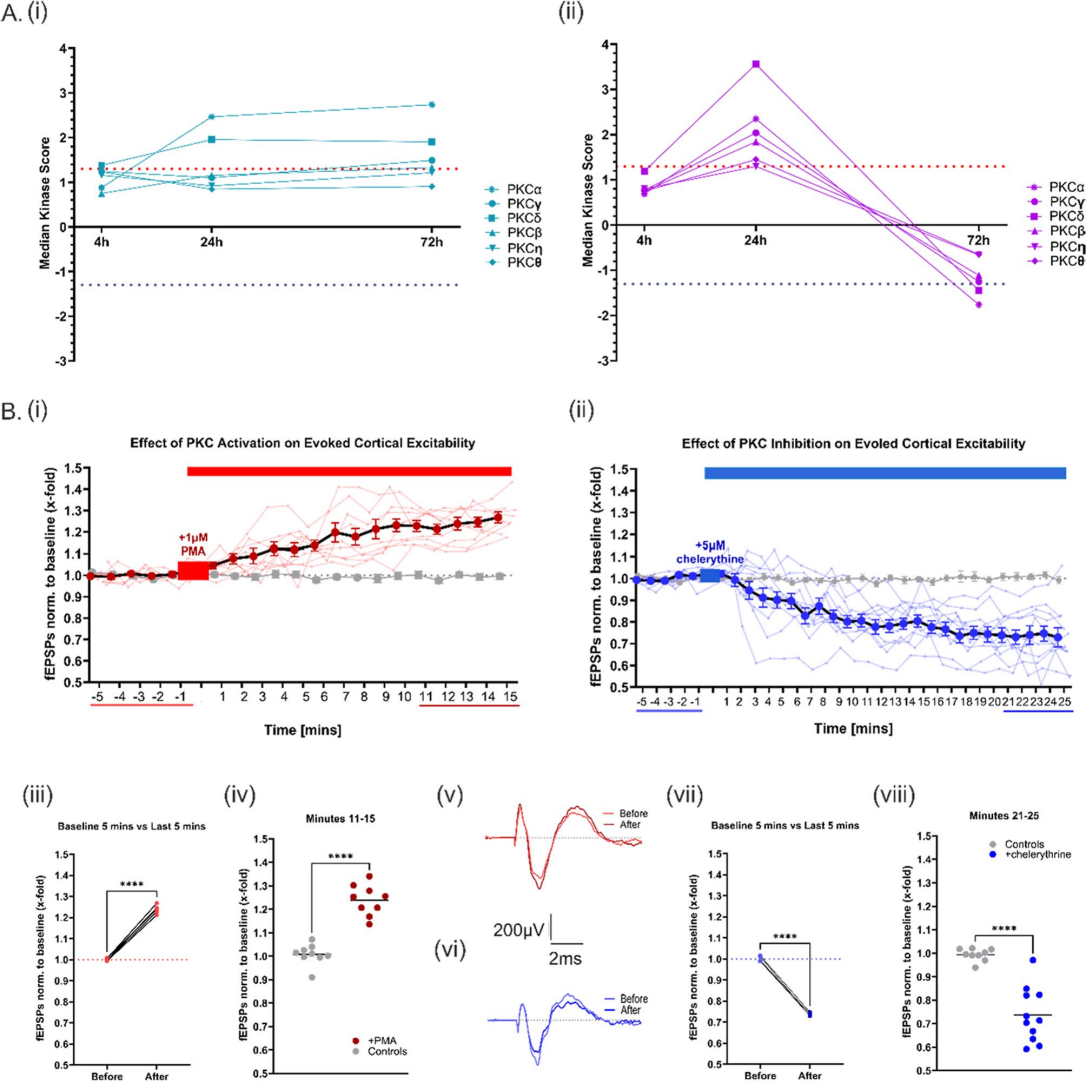
Dynamic PKC Activity Changes have Significant Influences on Cortical Excitability. **A** (i) Temporal profile of PKC kinase family activity from 4 to 72 h post-TBI in the ipsilateral cortical hemisphere. (ii) Temporal profile of PKC kinase family activity from 4 to 72 h post-TBI in the contralateral cortical hemisphere. **B** (i) PKC-activating PMA wash-in experiment showing that PKC activation leads to an increase in evoked EPSPs (n = 9). (ii) PKC-inhibiting chelerythrine wash-in experiment showing that PKC inhibition leads to a decrease in evoked EPSPs (n = 11) (iii) There is a significant increase in evoked EPSP amplitude when comparing the 5 min previous to PMA wash-in versus the last 5 min (minute 11–15) of the recording following PMA wash-in. (iv) There is a significant increase between minute 11–15 of the control recordings (no drug wash-in, n = 9) versus minute 11–15 of the recordings following PMA wash-in. (v) Exemplary fEPSP sweeps of before and after PMA wash-in. (vi) Exemplary sweeps of before and after chelerythrine wash-in. (vii) There is a significant decrease in evoked EPSP amplitude when comparing the 5 min previous to chelerythrine wash-in versus the last 5 min (minute 21–25) of the recording following chelerythrine wash-in. (viii) There is a significant increase between minute 21–25 of the control recordings (no drug wash-in, n = 9) versus minute 11–15 of the recordings following chelerythrine wash-in. Statistical analysis for before versus after drug wash-in was carried out using paired t-test. Statistical analysis for baseline recording versus last five minutes of drug wash-in was carried out using unpaired t-test. p < 0.05

PKC is a kinase that is unique in the AGC family in that it makes an emergence at the 24-h post-TBI timepoint in both hemispheres (particularly so in the contralateral hemisphere), to then continue into a hemisphere-specific activity profile by the 72-h timepoint, as shown in Fig. 4A, i, ii. This dynamic PKC activity profile in the contralateral hemisphere led us to wonder if the high PKC activity at the 24-h timepoint followed by the reduction at the 72-h timepoint was relevant to network activity in the contralateral hemisphere post-TBI. To determine this, after 10 min of baseline recording, 1 µM of the PKC-activating drug PMA was washed in via bath-application to the slice while the slice was being stimulated with 2.5 mV once every minute. As shown in Fig. 4B, i, the activation of PKC by PMA caused an increase in the amplitude of evoked field excitatory postsynaptic potentials (fEPSPs). Figure 4B, iii, iv shows that this increase in amplitude was significant, both when compared to the pre-wash-in baseline activity (minute − 5 to − 1 in graph) and to the control recordings which had no drug wash-in (grey data points in graph). Figure 4B, v shows a representative trace of an evoked fEPSP before and after the wash-in of PMA. In order to also pharmacologically mimic the significant decrease in PKC activity at the 72-h post-TBI timepoint in the contralateral hemisphere, and investigate the effects this would have on the network excitability, a further wash-in experiment using 5 µM of the PKC-inhibiting drug chelerythrine was carried out, as seen in Fig. 4B, ii. Conversely to the effect of the activation of PKC, the inhibition of PKC caused a decrease in the amplitude of the evoked fEPSPs. Again, this decrease in amplitude was significant, both when compared to the pre-wash-in baseline activity (minute − 5 to − 1 in graph) (Fig. 4B, vii) and to the control recordings which had no drug wash-in (grey data points in graph) (Fig. 4B, viii).

A representative trace of an evoked fEPSP before and after the wash-in of chelerythrine is shown in Fig. 4B, vi. These experiments verified the influence that PKC (in)activity does indeed have on cortical network excitability, creating another link between induced kinase activity changes and their electrophysiological functional implications after TBI.

### Inhibition of PKC activity rescues the hyperexcitability induced in the contralateral cortical hemisphere at 24 h post-TBI

**Fig. 5:**
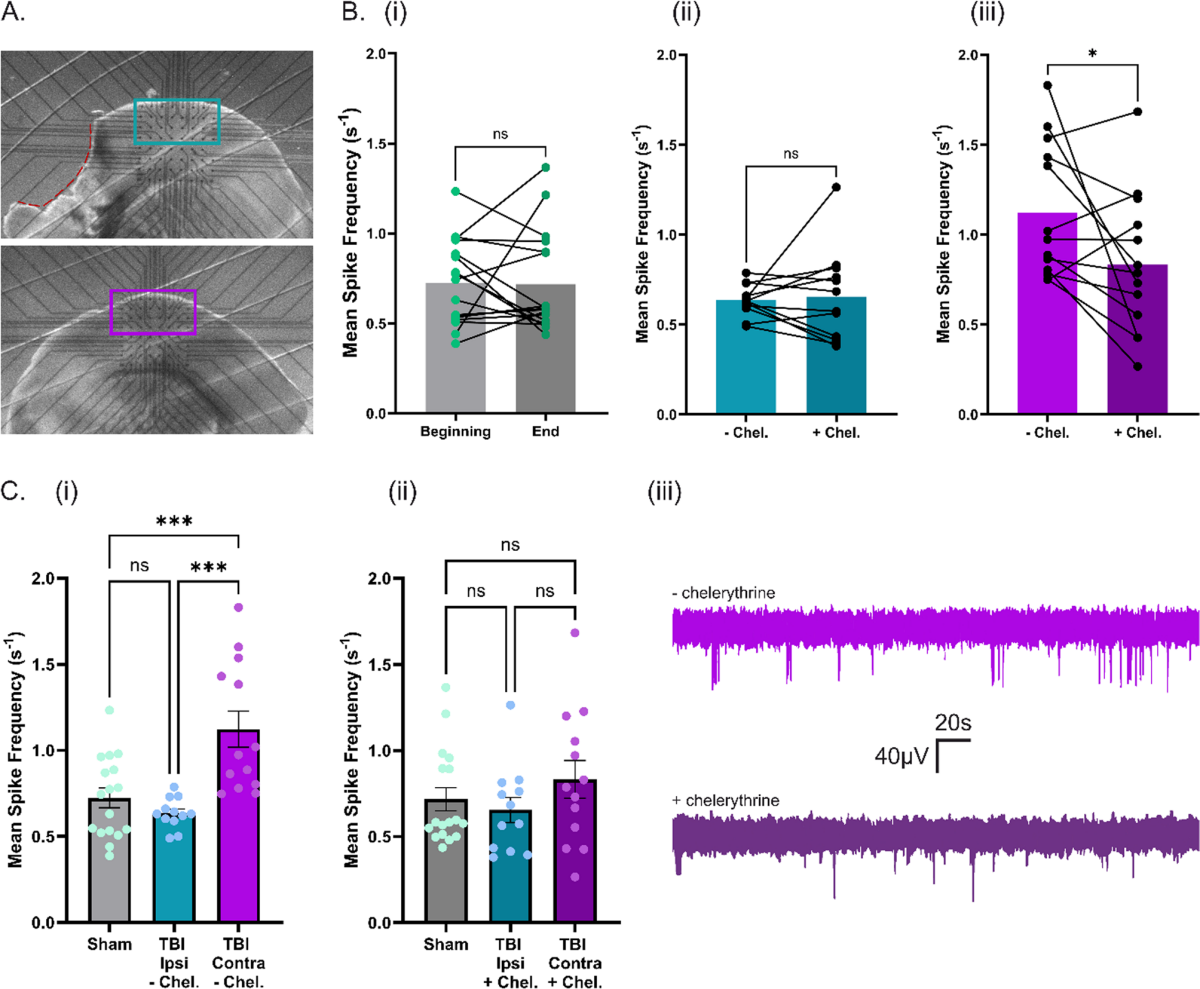
Inhibition of PKC Activity Rescues the Increased Spontaneous Activity of the 24-h post-TBI Contralateral Cortical Network back to Sham Level. **A** Representative slices from the ipsilateral (top) and contralateral (bottom) hemispheres on the MEA chip, with the recording electrodes outlined by the turquoise and purple rectangle, respectively. **B** (i) Mean spike frequency of the sham cortical hemisphere at the beginning and end of the MEA recording duration. (ii) Mean spike frequency of the post-TBI ipsilateral hemisphere before and after the 25-min chelerythrine wash-in period. (iii) Mean spike frequency of the post-TBI contralateral hemisphere before and after the 25-min chelerythrine wash-in period. **C** (i) Sham, ipsilateral and contralateral hemisphere mean spike frequency before the wash-in of chelerythrine. (ii) Sham, ipsilateral and contralateral hemisphere mean spike frequency after the wash-in of chelerythrine to the post-TBI slices. (iii) Representative five-minute traces of the spontaneous spiking activity from the same electrode before (light purple) and after (dark purple) the wash-in of chelerythrine to the 24-h post-TBI contralateral hemisphere. Statistical analysis of each hemisphere group before and after chelerythrine application/25-min recording duration was carried out using paired t-test. Statistical analysis comparing the three groups before and the three groups after the chelerythrine wash-in to post-TBI slices was carried out using one-way ANOVA and post hoc Tukey multiple comparison test. p < 0.05

Now with the knowledge of how the active kinome is temporally evolving after TBI, as well as how 24-h post-TBI kinase (in)activities influence the excitability of the cortical network, we next wanted to apply this to rescuing the electrophysiological alterations that occur at this timepoint; i.e., the cortical network hyperexcitability at 24 h post-TBI in the contralateral hemisphere [36, 39]. ERK1/2 does not have a specific activator and so the pharmacological activation of ERK1/2 to counteract its significant decrease in activity/expression at 24 h post-TBI could not be done. The pharmacological inhibition of PKC activity is possible, however, and so we used 5 μM chelerythrine to inhibit the significant overactivity of PKC which occurs at 24 h post-TBI. Coronal slices from 24-h post-TBI brains were placed on the MEA chip as described previously, and recordings taken from the ipsilateral (Fig. 5A), turquoise), contralateral (Fig. 5A), purple) or sham-operated hemispheres. Spontaneous activity was recorded via mean spike frequency from five-minute traces, with the first recordings being taken before the wash-in of chelerythrine. Following the 25-min wash-in period of chelerythrine to the post-TBI hemispheres, a second five-minute spike frequency trace was recorded. As seen in Fig. 5B, the addition of chelerythrine significantly reduced the mean spike frequency in the contralateral (iii) hemisphere. Interestingly, no change was found in the spiking rate of the ipsilateral (ii) hemisphere. To confirm whether or not the inhibition of PKC activity in the 24-h post-TBI contralateral hemisphere does indeed have a rescuing effect, we compared all three groups from before and after the addition of chelerythrine. Here, the significant hyperexcitability of the contralateral hemisphere compared to both the sham and ipsilateral hemisphere was confirmed (Fig. 5C, i). After the wash-in of chelerythrine, all three hemispheres displayed a comparable mean spike frequency (Fig. 5C, ii). These results indicate that the manipulation of PKC activity through pharmacological inhibition not only decreases the contralateral hemisphere hyperexcitability seen at the 24-h timepoint post-TBI, but also rescues the spontaneous activity back to sham level.

### Peptide level changes are indicative of the upstream kinase activity mechanisms which lead to hyperexcitability

**Fig. 6:**
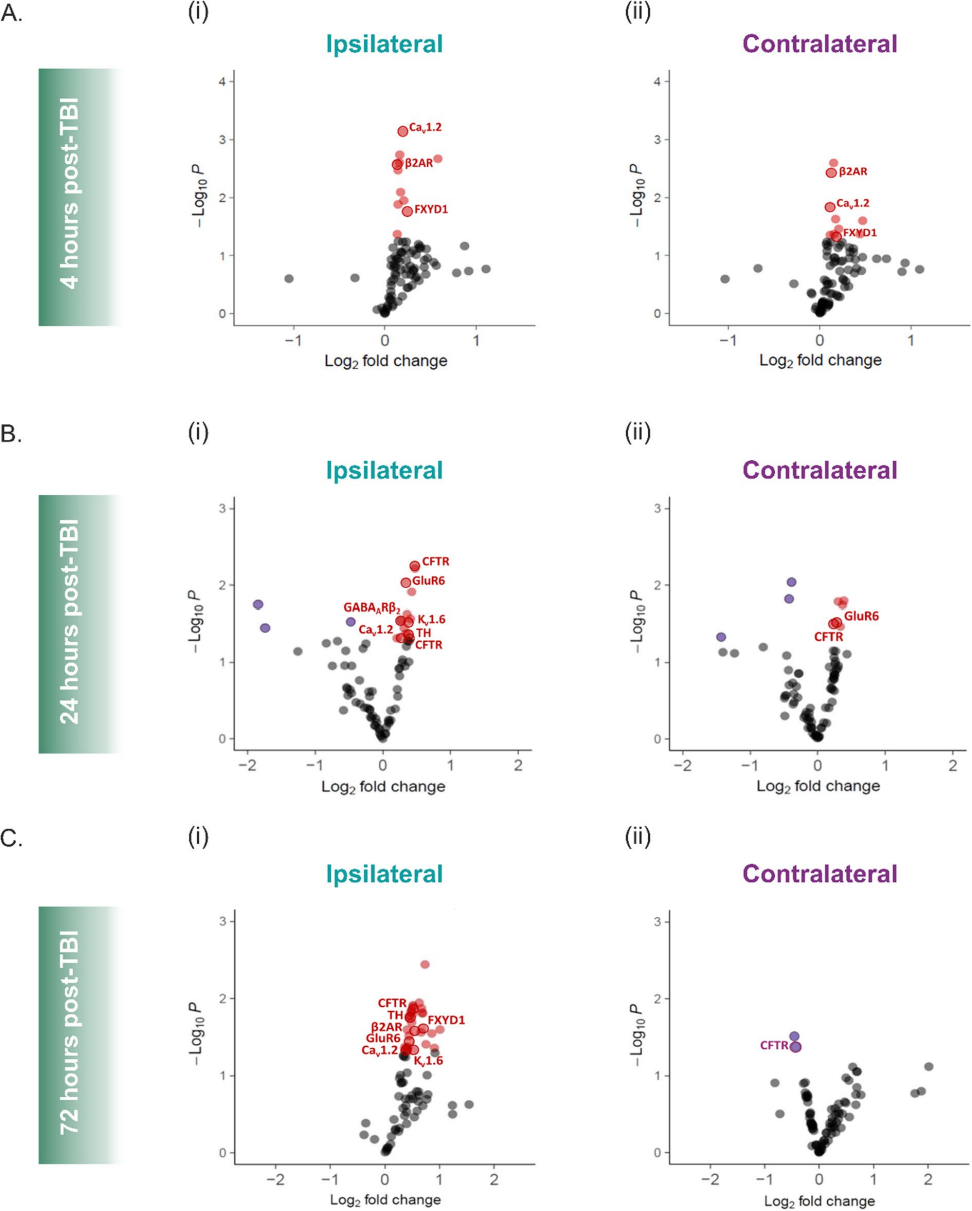
Significantly Over-/Under- Phosphorylated Peptide Targets at each Timepoint are Indicative of the Underlying Mechanisms Involved in post-TBI Network Hyperexcitability. Peptides associated with neurotransmission and/or excitability are labelled. **A** At 4 h post-TBI, the voltage-gated L-type calcium channel subunit α Cav1.2, the β2 adrenergic receptor (β2AR) and the FXYD domain-containing ion transport regulator 1 (FXYD1) are significantly over-phosphorylated in both the ipsilateral (i) and contralateral (ii) hemisphere. **B** (i) Cav1.2, ionotropic glutamatergic kainate receptor 2 (GluR6), GABA_A_ receptor β2 subunit (GABA_A_Rβ2), cystic fibrosis transmembrane conductance regulator (CFTR), tyrosine hydroxylase (TH) and potassium voltage-gated channel subfamily A member 6 (Kv1.6) are all significantly over-phosphorylated at 24 h post-TBI in the ipsilateral hemisphere. (ii) GluR6 and CFTR are significantly overphosphorylated at 24 h post-TBI in the contralateral hemisphere. **C** (i) Cav1.2, β2AR, FXYD1, GluR6, CFTR, TH and Kv1.6 are significantly overphosphorylated at 72 h post-TBI in the ipsilateral hemisphere. (ii) CFTR is significantly underphosphorylated at 72 h post-TBI in the contralateral hemisphere. All comparisons are relative to sham-operated control hemispheres at the same timepoint post-surgery. p < 0.05

In order to further understand the downstream targets that may be leading to the kinase activity-associated changes in network excitability, we examined the phosphorylation changes from the kinase assay at the peptide level. Figure 6 shows volcano plots for each cortical hemisphere at each timepoint post-TBI, with significantly hyperphosphorylated peptide substrates shown in red and significantly underphosphorylated peptide substrates shown in dark purple. The peptide substrates that relate to neuronal excitability (i.e., receptor or channel subunits, neurotransmitter-associated enzymes, etc.) are labelled. A list of all the significantly over-/under- phosphorylated peptide substrates, along with each associated Log2-ComBat-normalised heatmap of the integrated signals, can be found in Supplementary Fig. 2. Three peptides from the 24-h post-TBI lysates were also analysed via Western Blot for their phospho-/total- peptide ratio at the protein expression level, but showed only a trend towards over-phosphorylation. However, the assay substrates are directly phosphorylated by the kinases that are active in the post-TBI brain tissue homogenates, providing a superior and purpose-built approach for detecting peptide phosphorylation. This allowed us some insight to the mechanism by which kinases influence network excitability.

At 4 h post-TBI (Fig. 6A), the same three peptides are over-phosphorylated in each hemisphere. This further highlights the high level of synchronicity between hemispheres at this acute timepoint post-injury, as we also saw at the upstream kinase activity level. The peptides themselves also demonstrate the calcium-centric pathology and transmembrane ionic imbalance which is characteristic of this timepoint; the voltage-gated L-type calcium channel subunit α Ca_v_1.2, the β2 adrenergic receptor (β2AR) and the FXYD domain-containing ion transport regulator 1 (FXYD1) are all increased in their phosphorylation. The modification of Ca_v_1.2 via phosphorylation as well as its receptor-mediated effects through its relationship with the β2AR imply a strong neuronal excitability at this timepoint. Knowing that supraphysiological calcium influx post-injury is one of the main driving forces of increasingly detrimental secondary injuries, it is enlightening to see the significant up-phosphorylation of FXYD1, for example, which has ramifications on the efficiency of the membrane potential-maintaining sodium–potassium pump (Na + /K + -pump) [40]. This may be a compensatory measure with the objective of attempting to restore the transmembrane ionic balance. Outside of these electrophysiological-associated peptides, we also note some inflammatory and neurodegenerative mediator changes at this acute point. For example, the anti-apoptotic effect of the phosphorylation of BCL2-associated agonist of cell death (BAD) in the contralateral hemisphere, as well as the pro-apoptotic effect of pro-mitotic Aurora Kinase A (AurA) phosphorylation in the ipsilateral hemisphere (Supplementary Fig. 2).

By 24 h post-TBI (Fig. 6B), as again with the upstream kinase activity, the hemispheres maintain some level of synchronicity but are now exhibiting some disparate hemispheric changes. This timepoint post-TBI contains both kinase activity changes which, when mimicked, resulted in significant network hyperexcitability. It is also the timepoint at which the contralateral hemisphere displays a hyperexcitability via mean spike frequency. In the ipsilateral hemisphere, the Ca_v_1.2 channel is still significantly phosphorylated, indicating a continued amelioration of calcium flux in the lesioned hemisphere. In the contralateral side, there is no longer a Ca_v_1.2 increase in phosphorylation, suggesting a faster return to baseline calcium activity in the contralateral hemisphere relative to the ipsilateral. Also, at this timepoint, there is an emergence of phosphorylation-dependent modifications in receptors aside from calcium channels, e.g., the ionotropic glutamatergic kainate receptor 2 (GluR6), which is increased in phosphorylation in both hemispheres at 24 h post-TBI. The phosphorylation of GluR6 on serine/threonine residues indicates an enhanced function, i.e., an increase in excitatory glutamatergic signalling and increased influx of calcium and sodium ions to the cell. As well as this boost in excitatory transmission, there is also an indication of suppressed γ-aminobutyric acid (GABA)-ergic inhibitory transmission via the phosphorylation of the GABA_A_ receptor (GABA_A_R) β2 (GABA_A_Rβ2) subunit in the ipsilateral hemisphere. Upon stimulation and phosphorylation of the GABA_A_R, often associated with PKC activity, there is a run-down of the receptor via the β2 subunit [41–47]. This decreases the chloride influx to the neurons and can reduce membrane surface expression of the receptor, leading to an overall decrease in GABAergic inhibitory transmission. A loss or reduction of powerful interhemispheric GABAergic inputs from the lesioned hemisphere to the contralateral hemisphere is a widely recognised issue following a unilateral brain injury [48–51]. This phosphorylation-dependent mechanism may be a strong candidate for a contributing underlying mechanism to the contralateral hyperexcitability, or even a potential therapeutic target. There is also an up-phosphorylation in both hemispheres of the cystic fibrosis transmembrane conductance regulator (CFTR); an anion channel whose activation plays a neuroprotective role against glutamate-induced excitotoxicity and neuronal cell death [52, 53]. The CFTR channel activity may be helping to reduce the oxidative stress which is induced by the stabilisation of inducible 6-phosphofructo-2-kinase/fructose-2,6-biphosphatase 3 (iPFK-2) by its own over-phosphorylation (Supplementary Fig. 2) under hypoxic conditions. The rate-limiting enzyme tyrosine hydroxylase (TH), which plays a critical role in the reactions required for the biosynthesis of the excitatory transmitters dopamine and norepinephrine, shows a significant increase in phosphorylation in the ipsilateral hemisphere. An acute increase in production and release of catecholamines may result in excitotoxicity of cortical cells in the lesioned cortex, although its potential neuroprotective roles have also been defended [54, 55]. Cell death is also likely being encouraged by the phosphorylation of neutrophil cytosol factor 1 (NCF-1), also known as p47-phox, which is a phagocytic nicotinamide adenine dinucleotide phosphate (NADPH) oxidase organiser that leads to a surge in reactive oxygen species (ROS).

Finally, at the 72-h post-TBI timepoint (Fig. 6C), we see strong divergence between the ipsilateral and contralateral hemispheres. At the peptide level, the contralateral hemisphere is calming; there are now only two peptides undergoing a significant change in phosphorylation in the assay, both of which are under-phosphorylated. This demonstrates a return of the peptide substrate modifications of the contralateral hemisphere to near baseline conditions by this timepoint. The ipsilateral hemisphere is instead undergoing a plethora of increased phosphorylation changes. The receptors/channels of the assay, or their respective subunits, continue being targets for phosphorylation. All previously mentioned electrophysiologically-relevant peptides (with the notable exception now of GABA_A_Rβ_2_)—Ca_v_1.2, β2AR, GluR6, Kv1.6, TH, FXYD1 and CFTR—are up-phosphorylated. Inflammation- and neurodegeneration- associated peptide substrates comprise a large proportion of the up-phosphorylated kinase targets in the ipsilateral hemisphere at this timepoint. For example, macrophage nuclear factor NFkB p105 subunit (NFkB1), superoxide anion-creator NCF-1, macrophage colony stimulating factor receptor (CSF1R) and cAMP response element-binding protein (CREB) (Supplementary Fig. 2). This consecutive wave of inflammation seen at the 3-day post-injury timepoint is well described as being a result of CDK-associated residential astrocyte and microglial activation and response [56–58].

## Discussion

The post-TBI kinome uncovered throughout this project revealed much more to us than just the activity levels of individual kinases. Understanding the implications of certain kinase activity, as well as the circumstances under which they would become over- or under- active, is a complex story in itself. To gain a temporal awareness of the whole active serine/threonine kinome at once allowed new understanding of how dynamic the post-TBI molecular activities really are, which aids further comprehension of the functional implications of such (in) activities. Although past studies have described the activation of specific kinases following TBI [59–66], including or excluding the temporal component [63, 67–69], the bigger picture in terms of coinciding and dynamically evolving activity of several other kinases is often abandoned. The functional implications of TBI-induced kinase activity has been examined in the past; it was explored either at the downstream levels of behaviour/cognition [59–62, 70, 71] or cell death/survival [62, 65, 72–74], rather than directly at the electrophysiological level. Still, they allow a ratification of our own results, as they have also shown alterations in ERK and AGC kinase activation following TBI, for example [63, 66, 69, 74]. Moreover, most previous literature also noted these alterations in the serine/threonine kinases—as opposed to the tyrosine kinases. We believe that our results, which focuses more around the occurrences that are upstream of neurocognitive modulation and neurodegeneration—i.e., neuronal activity-dependent signalling and electrophysiological traits—can add a further and rounded understanding to this topic.

Even while the ipsilateral and contralateral hemispheres display a high degree of similarity in their kinomic profile, it is in their subtle differences that afford us details of hemisphere-specific qualities. Although at first some these changes may seem unrelated to network excitability, they actually play a prominent role for our understanding of why each hemisphere has its own electrophysiological profile regardless of periodic kinomic synchronicity. One example of these subtleties is the overarching pro-apoptotic forces in the ipsilateral hemisphere that were contrary to the generally neuroprotective and antiapoptotic mechanisms in the contralateral hemisphere. At the most acute stage, when hemispheric synchronicity is seen to be at its highest, there was only one single CAMK member—positive PCD regulator, DAPK2—that had a differential overactivity in the ipsilateral hemisphere only. At this same timepoint at the peptide level, AurA is only significantly up-phosphorylated in the ipsilateral hemisphere, which would imply a pro-apoptotic effect via noxious pro-mitotic processes in the post-mitotic neuronal cells. With similar abhorrent pro-mitotic indications, we can also consider CDKs. CDK overactivity is exclusive to the ipsilateral hemisphere in five cases out of the seven CDKs that were found to be differentially overactive in either hemisphere at 4 h post-TBI, as well as three out of the four differentially active CDKs at 72 h post-TBI (juxtaposed to the activity of those same three CDKs in the contralateral hemisphere at the 72-h timepoint, which are barely detectable). Contrarily, the significantly reduced phosphorylation of mitosis-associated peptides at 24 h post-TBI in the contralateral hemisphere, e.g., histone H3.2, M-phase inducer phosphatase 1 (cdc25A) and Ets2 repressor factor (ERF), would in fact imply the prevention of apoptosis, at least through pro-mitotic processes [75, 76]. Also, the significant up-phosphorylation of BAD peptide (usually by Akt1) at 4 h post-TBI in the contralateral hemisphere only, has anti-PCD effects [77].

Apart from the more obvious pro-apoptotic indications, it was also interesting to note the inflammatory mediator changes over time. At the 4-h post-injury point, the crucial upstream player in the NFkB signalling pathway—IKK—is actually more significantly overactive in the contralateral hemisphere, not the ipsilateral hemisphere. This is contrary to the 72-h timepoint whereby IKK complex subunits are only significantly overactive in the ipsilateral hemisphere and not past-threshold at all in the contralateral hemisphere. This kinase activation is reflected at the peptide level by the significant up-phosphorylation of the NFkB p105 subunit in the contralateral hemisphere at 4 h and then in the ipsilateral hemisphere at 72 h. Considering the substantial differences between the lesioned and the undamaged hemisphere at these two timepoints, and that this signalling pathway regulates transcription of both pro- and anti- inflammatory genes, we considered a hemisphere-specific effect of the pathway. In fact, this intriguing response has been seen in previous studies that delved into the inflammatory and synaptic activity changes induced by transcription factor pathway upregulations, like NFkB, proceeding a unilateral brain injury [78]. Another study which focused on gene expression and the immune response in both the ipsilateral and contralateral hemisphere after a unilateral cortical injury also divulged a baseline immune response in both hemispheres on account of the unilateral injury, wherein the contralateral hemisphere gene expression was on the anti-inflammatory axis while the ipsilateral hemisphere was on the pro-inflammatory axis [79]. This was also corresponding with the fact that macrophage activation was present in the ipsilateral hemisphere, but not the contralateral [79], which has also been previously confirmed in our own lab [36]. We can also see this at the peptide level of our kinase assay, which displayed a significant up-phosphorylation of the macrophage colony stimulating factor receptor (CSF1R) in the ipsilateral hemisphere at 72 h post-TBI, which is not significantly phosphorylated at this or any other timepoint in the contralateral hemisphere after injury.

The ipsilateral hemisphere cell death that occurs first as necrosis and later as apoptosis in the lesioned tissue is of course detrimental to brain health [17, 80]. Preventing neuronal cell apoptosis following a TBI is a primary objective by therapeutic candidates [81–83]. Despite the overarching apoptotic forces in the ipsilateral hemisphere being harmful in the way of secondary injury progression and maladaptive cortical network reorganisation, it may also be the reason why the ipsilateral hemisphere does not experience a pernicious network hyperexcitability at the 24-h timepoint. At 24 h post-TBI, a network hyperexcitability which was exclusive to the contralateral hemisphere has been observed in our lab, both previously [36, 39] and now again confirmed during this study (Fig. 5C, i). The kinase activity changes found during this project at this same timepoint of 24 h also caused a cortical hyperexcitability when pharmacologically mimicked in vitro. But, the kinome of the ipsilateral and contralateral hemisphere at 24 h—as we saw through the kinase assay—share a high degree of similarity. Why, then, does the ipsilateral cortical network not also display a hyperexcitability at 24 h after injury? We entertain two hypotheses for this.

The first considers that this is due to the rampant cell death which is present in the ipsilateral hemisphere and not in the contralateral hemisphere, subsequently leading to the functional breakdown of the ipsilateral cortical network. Since the contralateral hemisphere—which is not mechanically damaged—is chiefly undergoing pro-survival and neuroprotective mechanisms, the network connectivity and functionality is intact. This means that the hyperexcitability-encouraging kinase activity changes occurring at this time can be realised. Meanwhile, the ipsilateral network is barely in a condition to function at all, never mind to realise this hyperexcitable state.

Our second hypothesis takes the PKC family isoforms into account. Although both hemispheres experience a general increase in PKC activity at 24 h post-TBI, the ipsilateral hemisphere only sees a significant overactivity in two of six PKC isoforms: PKCα and PKCδ. The contralateral hemisphere instead has a significant overactivity in all six of the PKC isoforms at 24 h post-TBI (see: Fig. 3)—including, importantly, PKCγ: a PKC isoform which is exclusively expressed in neurons and not found anywhere else outside of the CNS [84–88]. Although PKCα and PKCδ are enriched in the brain (as are all PKC isoforms when compared to other tissue [87, 89]), they are not neuron-specific, and can also be active in glial and inflammatory cells [90–92]. To investigate post-TBI PKC isoform activity localisation, we carried out immunofluorescent staining comparing PKCα and PKCγ colocalization with NeuN and GFAP in the ipsilateral versus the contralateral hemisphere at 24 h post-TBI (Supplementary Fig. 6). This showed that PKCγ was strongly expressed at the neuronal cell membrane for both the ipsilateral and contralateral hemispheres, with the contralateral hemisphere showing a prominent expression through to layer 1 of the cortex (Supplementary Fig. 6, top panels). The expression localisation of PKCα, however, is much less defined. While some of this PKC isoform also colocalises with the neuronal cell membrane, it tended to be generally more scattered in its presentation (Supplementary Fig. 6, lower panels). This alludes to the more heterogenic character of PKCα activity, especially relative to the strongly neuronally-localised PKCγ activity. PKCα in the homotopic region of the unlesioned hemisphere is also very clear, but unlike PKCγ it does not spread through to layer 1. Another general point we can gather from these immunofluorescent stainings is that astrogliosis is vigorously attendant in the ipsilateral hemisphere and not in the contralateral hemisphere. Despite its presence, neither of the PKC isoforms appear to colocalise with these glia, but is likely expressed in other non-neuronal cells.

We deduced from this project just how difficult it can be to distinguish post-TBI beneficial and compensatory mechanisms from maladaptive or pathological ones. One primary example of this was the activation of AMPKα1, significantly so in the contralateral hemisphere at 24 h post-injury. Its activation is triggered when there is even a slight increase in the intracellular AMP/ATP ratio, acting in this way as an energy sensor for the cell to prevent an energy depletion that would otherwise inflict cellular stress [93]. Due to the harsh disruption of blood flow to the cortical cells post-injury, insufficient ATP is generated, causing secondary injuries ranging from mitochondrial dysfunction to Na^+^/K^+^-pump debilitation. As well as the reduced ATP production, there is also an increase in ATP demand, as the brain attempts to deal with the ionic imbalances, unconstrained neurotransmitter release and processes required for repair [94, 95]. The activation of the AMPK pathway by specific drug therapies has recently been gaining increased recognition for its neuroprotective implications against traumatic brain injury [98–101]. This seems positive, until we note that AMPK can also phosphorylate iPFK-2 [102, 103], which is indeed significantly up-phosphorylated at the peptide level of the kinase assay at this timepoint. iPFK-2 is induced under hypoxic conditions, such as TBI, and when phosphorylated it becomes stabilised and pushes the neurons towards glycolytic metabolism rather than the sustainable pentose-phosphate pathway (PPP) metabolism. Without the PPP in neuronal mitochondria, NADPH cannot be regenerated and therefore the redox balance in the cells cannot be maintained, leading to neurodegeneration [104]. iPFK-2 has even been targeted for inhibition in in vivo mouse ischaemic stoke models, as a method to alleviate neurotoxicity caused by overexcitation [105]. So, despite attempts towards saving cellular energy, this iPFK-2 phosphorylation by AMPK activation may lead to neuronal degeneration regardless. Then, in what appears as a subsequent effect, CFTR is also significantly up-phosphorylated at this timepoint. The activation of CFTR in such a scenario has been shown to reduce the redox distress that is produced from glutamate-induced excitotoxicity, even showing an inverse corelation between mitochondrial oxidative stress and CFTR activation, thereby protecting the neurons from cell death [52, 106]. Identifying and interpreting so many changes simultaneously after TBI was a large success of the kinase assay.

From looking at the temporal profiles of all serine/threonine kinases in their respective groups or families (Supplementary Fig. 3) it can be seen that for all kinases except for the AGC group, there is a notable lull in activity at the 24-h post-TBI timepoint. With the exception of a couple of CAMK members, the AGC group undergoes a period of strong overactivity while other kinases are either underactive or comparable to sham activity levels. Having no opposing kinase activity to counter that of high AGC activity may allow the group to have a stronger influence on the cortical hemispheres at this 24-h post-injury stage. That being said, we also now know that even significantly underactive kinases, e.g., ERK1/2, can evoke extreme effects on the functional network.

The significant electrophysiological response of inhibiting ERK1/2 remains obscure in terms of the underlying mechanism. As mentioned above, ERF – which is exclusively downstream of ERK1/2 and has even been used as an indicator for ERK activity [107] – is significantly underphosphorylated at 24 h post-TBI at the peptide level. Without phosphorylation, ERF remains in the cell nucleus and represses gene transcription important for cell cycle progression. When we look to the wash-in of the ERK-inhibitor FR180204 and the timing of the effect exertion (Supplementary Fig. 4), the maximum induced amplitude is reached at approximately 50 min. This timing could potentially align with timing of gene transcription [108], however more time would likely be necessary for translation of target genes. Future experiments should be undertaken to better understand the relationship between ERK activity and network excitability.

An interesting point regarding the peptide level changes from this kinase assay is the phosphorylation and subsequent rundown of the GABA_A_R via the β_2_ subunit, specifically in the ipsilateral hemisphere and only at 24 h post-TBI. As mentioned in the results above, this may be one explanation for the loss of powerful interhemispheric GABAergic inputs from the injured hemisphere to the contralateral hemisphere; a characteristic of unilateral injuries such as TBI or stroke. Together with the increased glutamatergic signalling, this has interesting links with the whole-cell patch-clamp recordings that have previously been done in our lab and show an increase in spontaneous α-amino-3-hydroxy-5-methyl-4-isoxazolepropionic acid (AMPA)/GABA ratio and a decrease in spontaneous inhibitory post-synaptic currents (sIPSCs) in both hemispheres at 24–48 h post-lesion [36]. It has been previously noted that phosphorylation-dependant oppression of GABAergic transmission can be regulated by PKC activation and its enhancement by PKC inhibition [47]. This is particularly interesting for us when we recall that PKC activity is indeed overactive at the 24-h post-TBI timepoint in our kinase assay. In our MEA experiments, pharmacological activation of PKC by PMA also increased network excitability, and inhibition decreased it. Further, the hyperexcitability of the contralateral cortical network at 24 h post-TBI could be reduced to sham level through PKC inhibition. Although the bath application method is not specific to one target, a PKC-governed phosphorylation of GABA_A_Rs may be a contributing underlying mechanism to this network excitability. Taken together, this phosphorylation-dependent reduction of interhemispheric GABAergic transmission which is induced after a brain injury is a potential therapeutic pathway that should be more closely investigated through future research.

It must also be addressed that this kinase assay was specific for serine/threonine kinase activity and does not account for the tyrosine kinases. Although a tyrosine kinase assay was attempted by us, the peptide level changes relative to sham controls were insufficient to deduce valid upstream kinase activity in the post-TBI ipsilateral and contralateral hemispheres. This is not to say that a post-TBI tyrosine kinase assay should not be again attempted in the future, as a higher sample number may already be sufficient to attain statistically valid results. In our case, the vast quantity of data that we were already able to surmise from this serine/threonine assay yielded several interesting research insights and potential therapeutic targets for TBI, leaving the tyrosine kinase assay for future work. Another potential limitation to this study is the fact that all experimentation was performed with an ex vivo/in vitro approach. Although we consistently take measures which ensure our results are as reliable and as close to the in vivo situation as possible, future experimentation which verifies our findings in a pure in vivo animal model would be of great interest.

Above all, the degree to which phosphorylation is involved not just in TBI but also in many other neurodegenerative and neurological diseases, is worth remarking. The pathology of Chronic Traumatic Encephalopathy (CTE) centres around phosphorylated tau (p-tau) [109], as does Alzheimer’s Disease (AD) [110]. In Parkinson’s Disease (PD), there is pathological increases in the phosphorylation of α-Synuclein (α-syn) [111], while hyperphosphorylation of TAR DNA-binding protein 43 (TDP-43) is a hallmark of amyotrophic lateral sclerosis (ALS) neuropathology [112]. The first steps in addressing perhaps some of our most monumentally challenging diseases could be simply describing, characterising and gaining a stronger understanding of the functional kinome.

## Conclusion

TBI induces numerous triggers for the activation of kinases. The functional changes that phosphorylation of downstream peptides by upstream kinases incur can be monumental. These targets include subunits of ion channels and receptors as well as regulators in neurotransmitter formation and ion pumps, amongst others. With dysregulation of network excitability being one of the leading and most problematic pathologies that emerges from TBI, it is undoubtedly important to characterise the post-TBI kinome and to understand the significant influence that these kinase activity changes have on the cortical network. With today’s therapeutic options for TBI being mostly ineffective or inflicting excessive side-effects, identifying targets which can potently bias the mechanism of key excitability regulators such as ion channels or receptors—without wholly agonising or antagonising their physiological role—should be more seriously considered.

## Supplementary Information

Below is the link to the electronic supplementary material.

Supplementary file1 (DOCX 10817 KB)

## Data Availability

The datasets generated during and/or analysed during the current study are not publicly available due to related ongoing research projects in our lab but are available from the corresponding author on reasonable request. Disclosure of Data Usage: Some of the data presented in this manuscript will also be included in the PhD thesis of author Celine Gallagher (under preparation) and will be cited as such. All data, methods, and findings are consistent with those presented in this manuscript, and proper citations will be provided in the final version of the thesis.

## Abbreviations

ACSF: Artificial cerebrospinal fluid
AD: Alzheimer’s disease
AGC: Kinase group primarily comprised of protein kinase A, G, C
AMPK: 5' Adenosine monophosphate-activated protein kinase
AurA: Aurora A kinase
BAD: Bcl-2-associated death promoter
CAMK: Ca2 + /calmodulin-dependent protein kinase
Cav1.2: L-type voltage-dependent alpha 1C subunit calcium channel
CCI: Controlled cortical impact
CCL: Chemokine ligand
CDK: Cyclin-dependent kinase
CFTR: Cystic fibrosis transmembrane conductance regulator
CREB: CAMP response element-binding protein
CSF1R: CSF1R colony stimulating factor 1 receptor
DAPK: Death-associated protein kinase
DMSO: Dimethyl sulfoxide
E/I: Excitatory/inhibitory
ECL: Enhanced chemiluminescence
ERK: Extracellularly-regulated kinase
FXYD: Highly conserved family of small membrane proteins with the amino acid sequence PFXYD
GABA(AR): Gamma aminobutyric acid (subtype A, receptor)
GluR6: Ionotropic glutamate receptor 6
HRP: Horseradish peroxidase
IKK: IkappaB kinase
IL: Interleukin
iPFK-2: Inducible 6-phosphofructo-2-kinase
JNK: C-Jun N-terminal kinase
Kv1.6: Potassium voltage-gated channel subfamily A member 6
LFC: Log fold change
LFP: Local field potential
MAPK: Mitogen-activated protein kinase
MEA: Multielectrode array
Na + /K + -pump: Sodium potassium pump
NADPH: Nicotinamide adenine dinucleotide phosphate
NCF-1: Neutrophil cytosol factor 1
NFkB: Nuclear factor ‘kappa-light-chain-enhancer’ of activated B-cells
NO: Nitric oxide
PCD: Programmed cell death
PKA: Protein kinase A
PKC: Protein kinase C
PKG: Protein kinase G or cGMP-dependent protein kinase
PMA: Phorbol 12-myristate 13-acetate
PRKX: Protein kinase X-linked
PTE: Post-traumatic encephalopathy
PTK: Protein tyrosine kinase
PTM: Post-translational modification
ROS: Reactive oxygen species
STK: Serine threonine kinase
TBI: Traumatic brain injury
TH: Tyrosine hydroxylase
UKA: Upstream kinase analysis
β2AR: Beta-2 adrenergic receptor
