# Correction: Temporal characterisation and electrophysiological implications of TBI-induced serine/threonine kinase activity in mouse cortex

**DOI:** 10.1007/s00018-025-05888-2

**Published:** 2025-09-24

**Authors:** Celine Gallagher, Thomas Mittmann

**Affiliations:** Institute of Physiology, University Medical Centre of the Johannes Gutenberg University Mainz, Mainz, Germany

**Correction: Cellular and Molecular Life Sciences (2025) 82:102**

10.1007/s00018-025-05638-4

In the original version of this article, the given and family names of "Celine Gallagher and Thomas Mittmann." were incorrectly structured. The name was displayed correctly in all versions at the time of publication.

The original article has been corrected.

