# Supplementary material for: Temporal characterisation and electrophysiological implications of TBI-induced serine/threonine kinase activity in mouse cortex
Source: Cell Mol Life Sci. 2025 Mar 5;82(1):102. doi: 10.1007/s00018-025-05638-4 (PMC11883073; doi:10.1007/s00018-025-05638-4)
Supplement: Supplementary file 1 — Supplementary file1 (DOCX 10817 KB) [file 18_2025_5638_MOESM1_ESM.docx]

# Supplementary Material


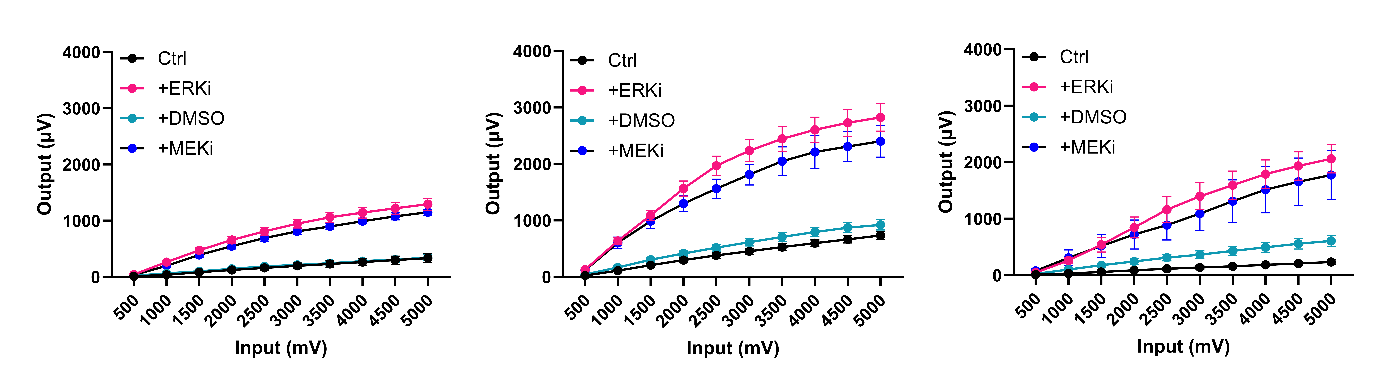


**Supplementary Figure 1. MEA Recordings to Control for the Drug Vehicle and ERKi.** There is no statistical difference between I/O curve of the drug vehicle DMSO alone versus ACSF alone without DMSO or drug. Inhibiting MEK, the sole upstream activator of ERK, has the same boosting effect on evoked fEPSPs as inhibiting ERK, verifying the effect.


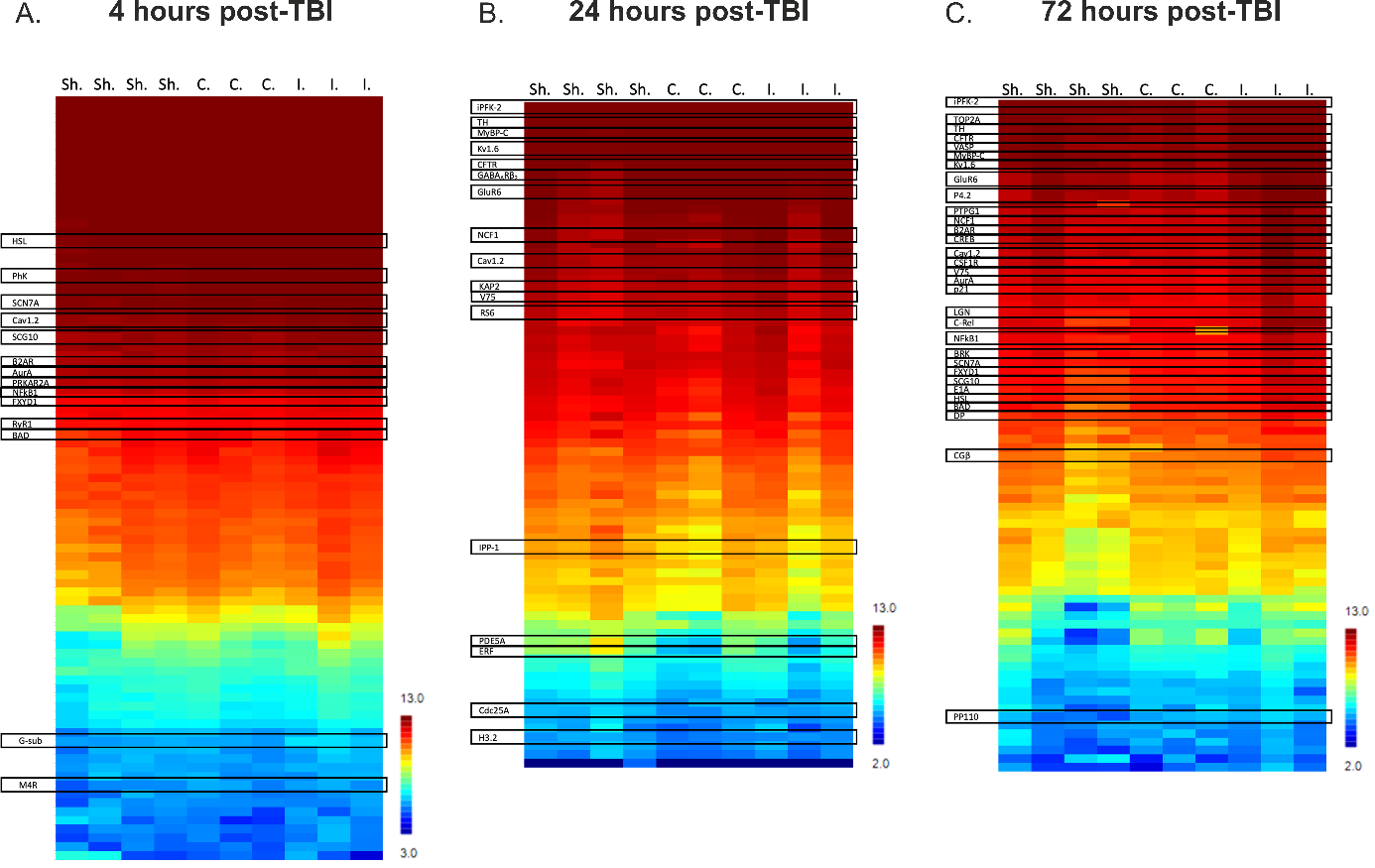


**Supplementary Figure 2. Log2-ComBat-normalised heatmap of the integrated signals from the Phosphosite Array for each Timepoint of Interest post-TBI.** **(A-C)** Labelled heatmap with legend identifying all significantly up-/down-phosphorylated peptides for 4 hours, 24 hours and 72 hours post-TBI.


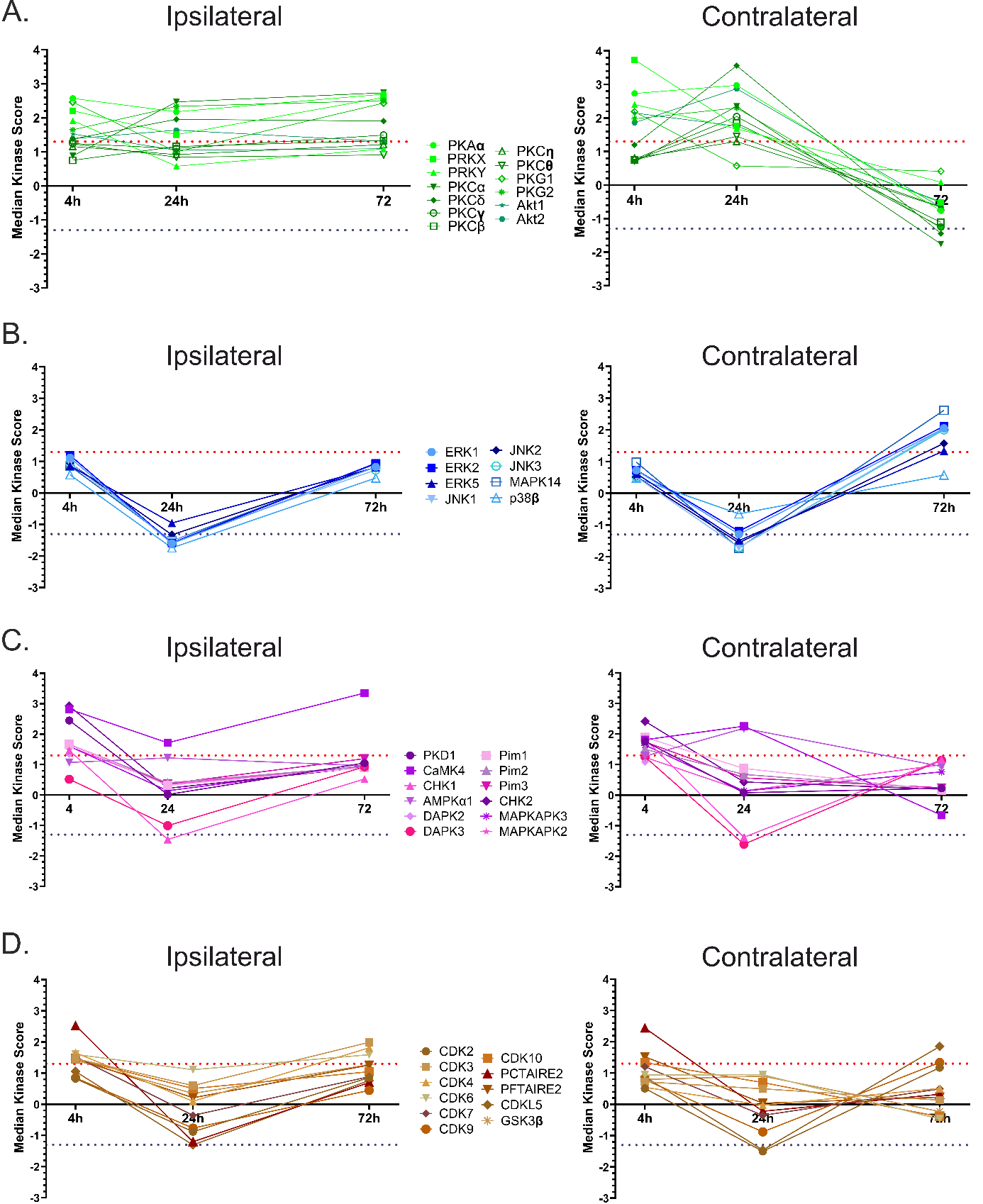


**Supplementary Figure 3. Temporal Overview of Kinase Activity Changes from 4 hours to 72 hours post-TBI.** **A.** Temporal kinase activity changes from 4 hours to 72 hours post-TBI in the AGC group. **B.** Temporal kinase activity changes from 4 hours to 72 hours post-TBI in the MAPK family. **C.** Temporal kinase activity changes from 4 hours to 72 hours post-TBI in the CAMK group. **D.** Temporal kinase activity changes from 4 hours to 72 hours post-TBI in the CDK & GSK family. Red line indicates threshold for significantly overactive kinase activity. Dark purple line represents threshold for significantly underactive kinase activity.


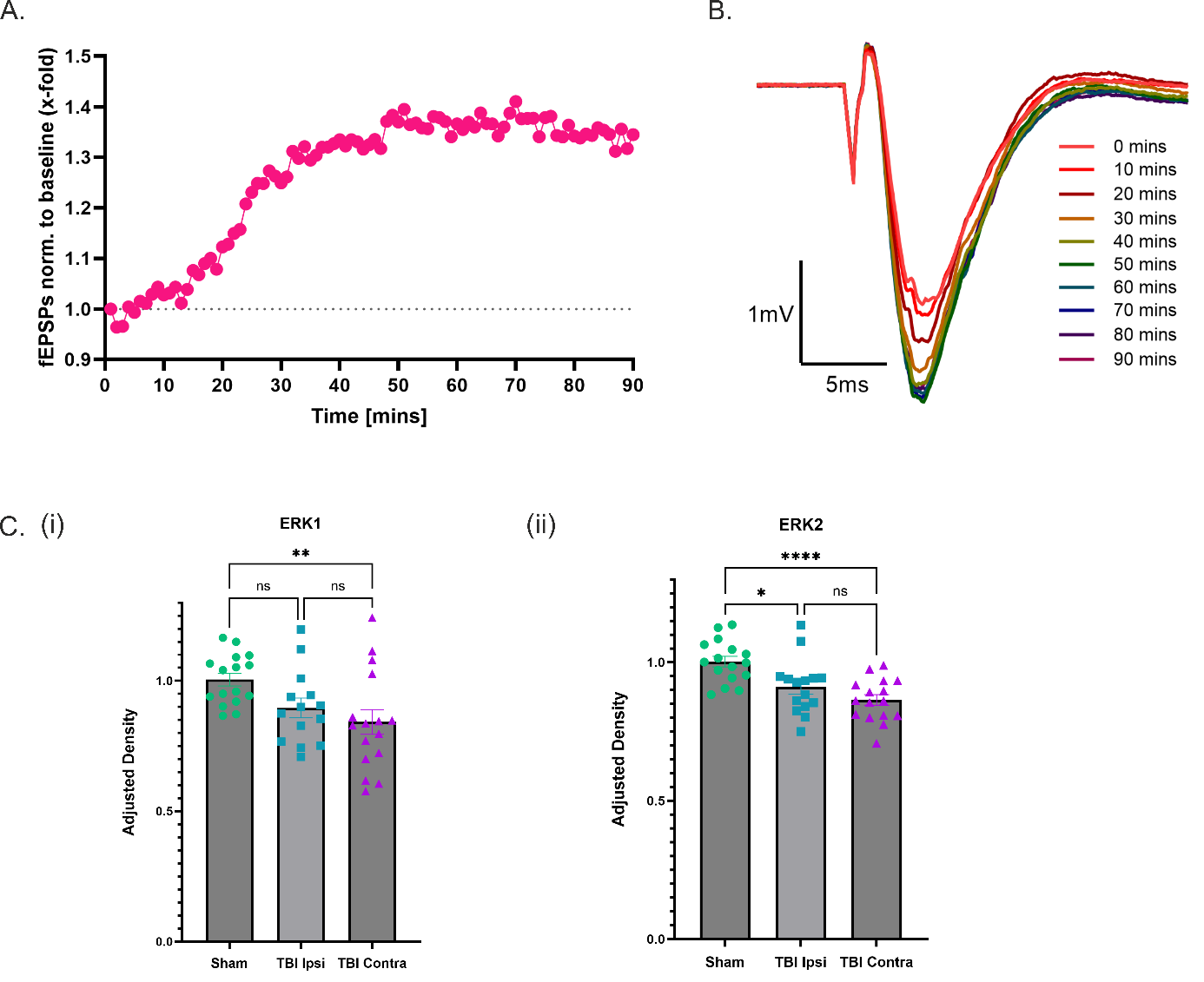


**Supplementary Figure 4. ERKi Wash-In and Continued Fluctuation of ERK expression at 1-week post-TBI. A.** Timing and maintenance of the effect of the ERK-inhibitor FR180204 on fEPSP signals. **B.** fEPSP traces from FR180204 wash-in. **C.** ERK expression again decreases at 1-week post-TBI, continuing its dynamic fluctuation of expression beyond 72 hours post-injury.


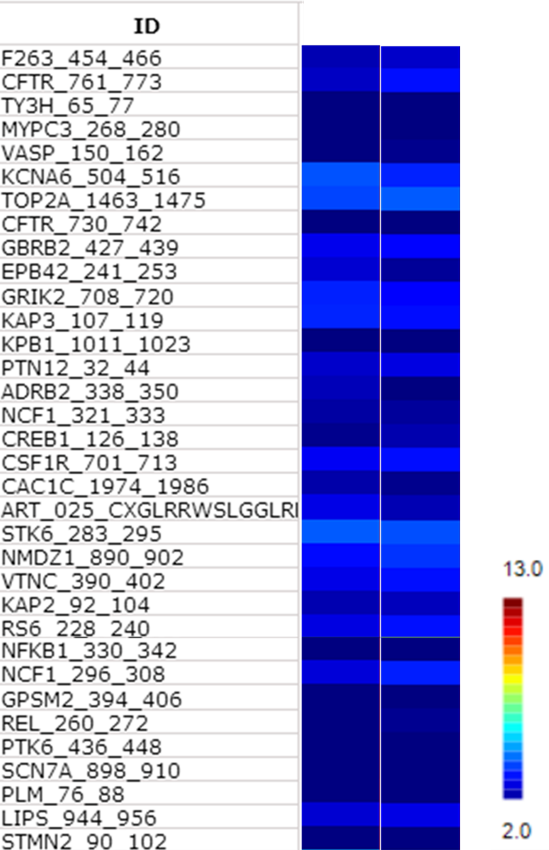


**Supplementary Figure 5. Control Log2-ComBat-normalised heatmap of the integrated signals from the Phosphosite Array for Lysis Buffer alone.** Lysis buffer alone without sample does not show any array peptide phosphorylation, as expected.


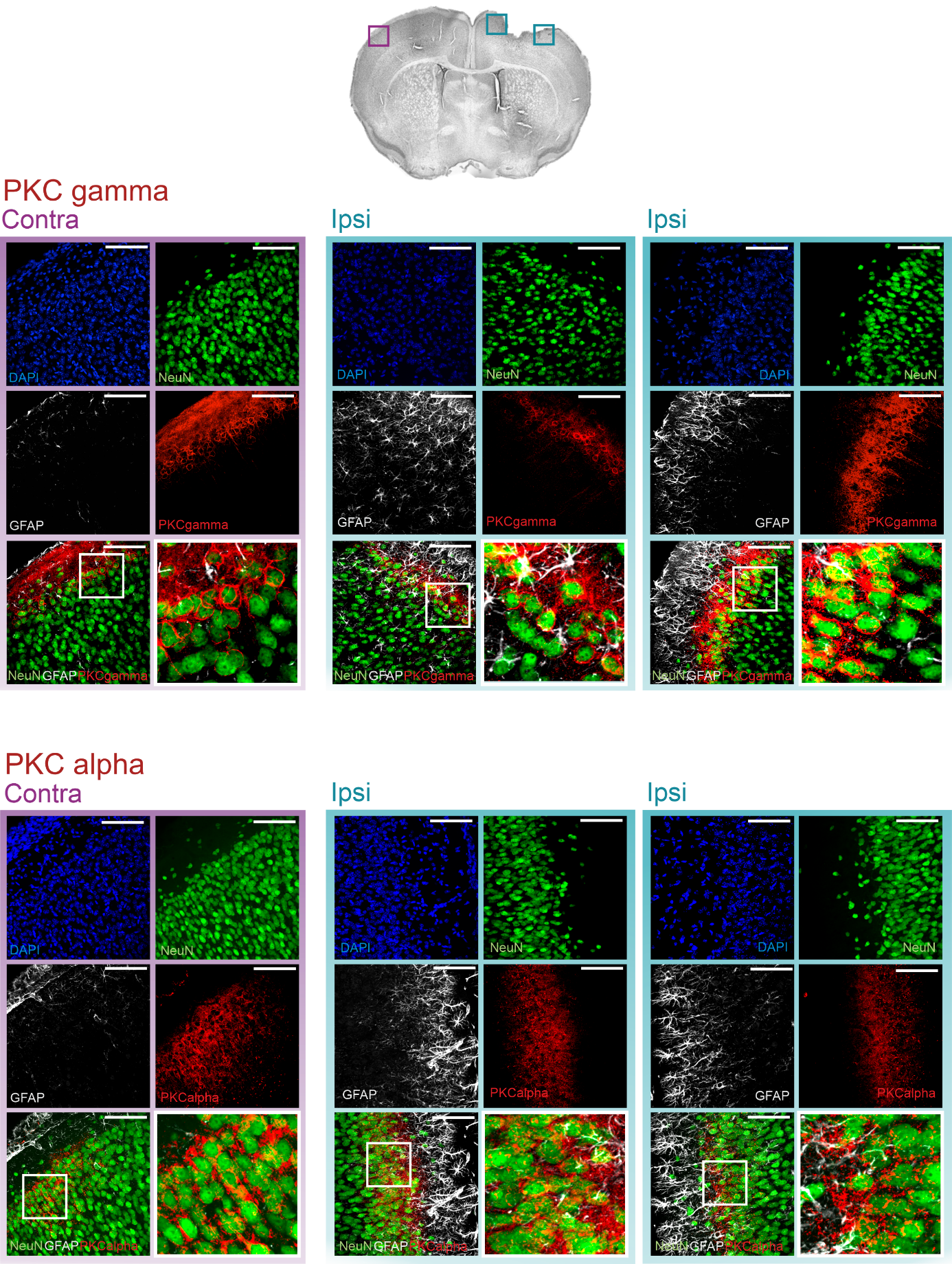


**Supplementary Figure 6. Immunofluorescent staining of PKCγ and PKCα colocalised with NeuN and GFAP in the 24-hour post-TBI Ipsilateral and Contralateral Cortical Hemispheres.** All stainings were first imaged with DAPI (blue) and then NeuN (green), GFAP (grey) and PKCγ (top panels, red) or PKCα (bottom panels, red) were colocalised to confirm cell-specific localisation of the PKC isoforms with either neurons or astrocytes. Scale bar = 100μm.


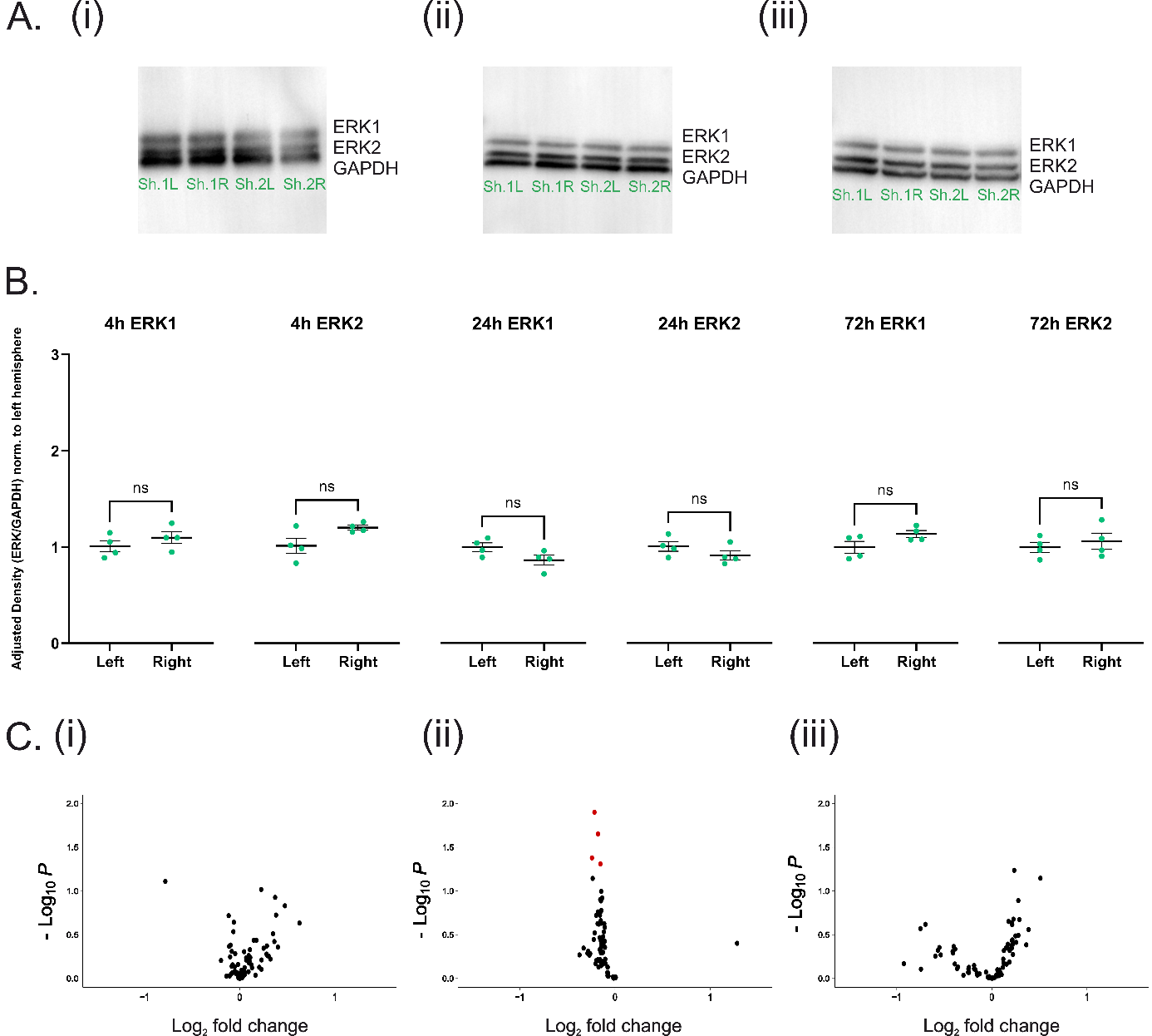


**Supplementary Figure 7. There is no difference between Sham Left and Sham Right cortical hemispheres at either protein expression level nor kinase activity level. A.** Western Blots showing ERK1/2 and housekeeper protein GAPDH at **(i)** 4 hours, **(ii)** 24 hours and **(iii)** 72 hours post-TBI for the sham hemisphere lysates. **B.** ERK1/2 expression levels at each timepoint of interest post-TBI showing no statistical difference (t-test analysis) between sham hemispheres. **C.** Comparison of sham left versus sham right cortical hemispheres in phosphorylating downstream peptide targets on the kinase assay array at **(i)** 4 hours, **(ii)** 24 hours and **(iii)** 72 hours post-TBI. Due to false discovery rate (FDR) for high-throughput experiments the minimum number of significantly altered phosphosites required for validation of statistical difference = 8; i.e., there is zero significance between sham hemispheres at any timepoint post-TBI.


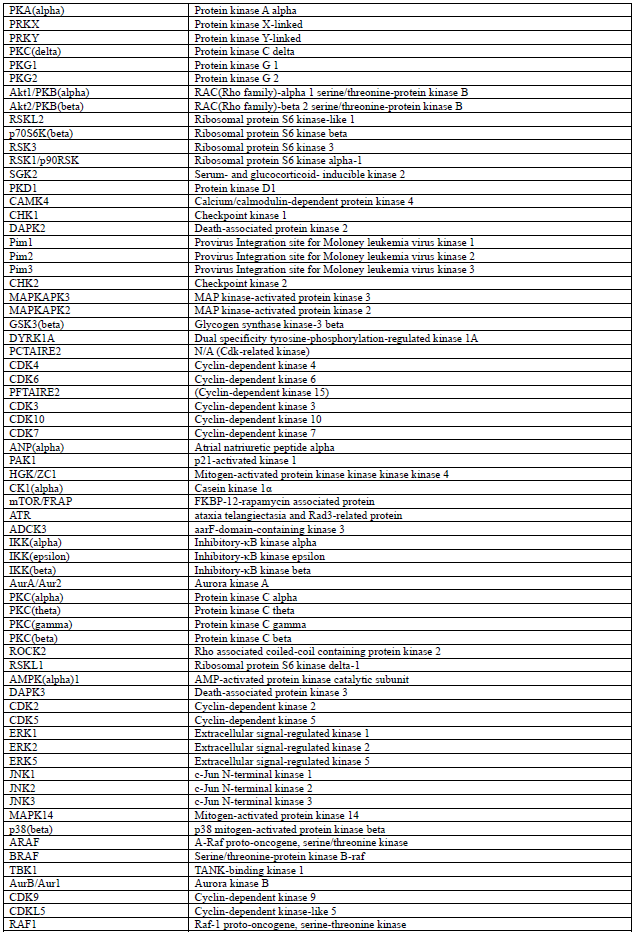
**Supplementary Table 1. Individual Kinase Abbreviations**
